# Supplementary material for: Impact of dietary aflatoxin on immune development in Gambian infants: a cohort study
Source: BMJ Open. 2021 Jul 20;11(7):e048688. doi: 10.1136/bmjopen-2021-048688 (PMC8292809; doi:10.1136/bmjopen-2021-048688)
Supplement: Author's manuscript [file bmjopen-2021-048688.draft_revisions.pdf]

BMJ Open

BMJ Open is committed to open peer review. As part of this commitment we make the peer review history of every article we publish publicly available.

When an article is published we post the peer reviewers' comments and the authors' responses online. We also post the versions of the paper that were used during peer review. These are the versions that the peer review comments apply to.

The versions of the paper that follow are the versions that were submitted during the peer review process. They are not the versions of record or the final published versions. They should not be cited or distributed as the published version of this manuscript.

BMJ Open is an open access journal and the full, final, typeset and author-corrected version of record of the manuscript is available on our site with no access controls, subscription charges or pay-per-view fees (http://bmjopen.bmj.com).

If you have any questions on BMJ Open's open peer review process please email info.bmjopen@bmj.com

BMJ Open

Impact of dietary aflatoxin on immune development in Gambian infants

Journal:	*BMJ Open*
Manuscript ID	bmjopen-2021-048688
Article Type:	Original research
Date Submitted by the Author:	05-Jan-2021
Complete List of Authors:	Xu, Ya; University of Leeds<br>Moore, Sophie; King's College London, Women and Children's Health<br>Chen, Gaoyun; Queen's University Belfast<br>Nshe, Patrick; MRC Unit The Gambia at LSHTM<br>Faye-Joof, Tisbeh; MRC Unit The Gambia at LSHTM<br>Prentice, Andrew; MRC Unit The Gambia at LSHTM<br>Gong, Yun Yun; University of Leeds<br>Routledge, Michael; University of Leeds, School of Medicine; Jiangsu University, School of Food and Biological Engineering
Keywords:	Epidemiology < TROPICAL MEDICINE, Nutrition < TROPICAL MEDICINE, IMMUNOLOGY

SCHOLARONE™
Manuscripts

I, the Submitting Author has the right to grant and does grant on behalf of all authors of the Work (as defined in the below author licence), an exclusive licence and/or a non-exclusive licence for contributions from authors who are: i) UK Crown employees; ii) where BMJ has agreed a CC-BY licence shall apply, and/or iii) in accordance with the terms applicable for US Federal Government officers or employees acting as part of their official duties; on a worldwide, perpetual, irrevocable, royalty-free basis to BMJ Publishing Group Ltd ("BMJ") its licensees and where the relevant Journal is co-owned by BMJ to the co-owners of the Journal, to publish the Work in this journal and any other BMJ products and to exploit all rights, as set out in our licence.

The Submitting Author accepts and understands that any supply made under these terms is made by BMJ to the Submitting Author unless you are acting as an employee on behalf of your employer or a postgraduate student of an affiliated institution which is paying any applicable article publishing charge ("APC") for Open Access articles. Where the Submitting Author wishes to make the Work available on an Open Access basis (and intends to pay the relevant APC), the terms of reuse of such Open Access shall be governed by a Creative Commons licence – details of these licences and which Creative Commons licence will apply to this Work are set out in our licence referred to above.

Other than as permitted in any relevant BMJ Author's Self Archiving Policies, I confirm this Work has not been accepted for publication elsewhere, is not being considered for publication elsewhere and does not duplicate material already published. I confirm all authors consent to publication of this Work and authorise the granting of this licence.

For peer review only

Impact of dietary aflatoxin on immune development in Gambian infants

Ya Xu[1], Sophie E. Moore[2, 3], Gaoyun Chen[4], Patrick Nshe[3], Tisbeh Faye-Joof[3], Andrew M Prentice[3], Yun Yun Gong[5], Michael N Routledge[1,6*]

[1]School of Medicine, University of Leeds, Leeds LS2 9JT, UK,

[2]Department of Women and Children's Health, King's College London, St Thomas' Hospital, 10th Floor North Wing, London SE1 7EH, UK,

[3]MRC Unit The Gambia at the London School of Hygiene and Tropical Medicine, Atlantic Boulevard, Fajara, PO Box 273, Banjul, The Gambia,

[4]Institute for Global Food Security, School of Biological Sciences, Queen's University Belfast, Belfast, UK,

[5]School of Food Science and Nutrition, University of Leeds, Leeds, UK.

[6]School of Food and Biological Engineering, Jiangsu University, Zhenjiang, Jiangsu Province, China

*Correspondence: medmnr@leeds.ac.uk

For peer review only

Abstract

Background Chronic aflatoxin (AF) exposure has been shown to occur at high levels in children from sub-Saharan Africa (SSA), and has been associated with growth retardation and immune dysfunction. Our objective was to investigate the impact of aflatoxin exposure on immune development in early infancy using thymic size and antibody (Ab) response to vaccination as indicators of immune function.

Methods A total of 374 infants born between May 2011 and December 2012 were enrolled into the current study. These infants were recruited from a larger, randomized trial examining the impact of nutritional supplementation of mothers and infants on infant immune development (the ENID Trial; ISRCTN49285450). Thymic size (Thymic Index; TI) was measured by sonography at 1, 8, 24 and 52 weeks of infant age. Infants were given the diphtheria-tetanus-pertussis (DTP) vaccine at 8, 12 and 16 weeks of age, and antibody responses to each vaccine measured at 12 and 24 weeks of age. Aflatoxin-albumin (AF-alb) adduct levels in infant blood were measured by ELISA as the biomarker of aflatoxin exposure.

Results The geometric mean (GM) level of AF-alb increased with age. Only half of infants had detectable AF-alb with a GM of 3.52 pg/mg at 24 weeks, increasing to 25.39 pg/mg at 52 weeks, when 98% of infants had AF-alb >limit of detection. Significant negative association of AF-alb level with TI were seen in infants during the first 24 weeks, especially at 8 weeks of age ($p<0.001$), which is the time point of fastest thymus growth. There were no associations

For peer review only - http://bmjopen.bmj.com/site/about/guidelines.xhtml

between aflatoxin exposure level and antibody response to pertussis and tetanus, but a significant positive correlation was observed between AF-alb level and antibody titre to diphtheria ($p<0.005$).

Conclusions High levels of aflatoxin exposure during early infancy may impact on infant immune development.

Keywords Aflatoxin, immune development, thymus, antibody response to vaccination

Strengths and limitations of this study

- The study was embedded within a clinical trial (ENID), which reduced selection bias of subjects
- The subjects were part of a cohort naturally exposed to aflatoxin through their diet, which allowed for multiple time point sampling
- The aflatoxin exposure was measured using a validated biomarker, which allows better estimation of exposure compared to food intake estimates
- A limitation of the study is that aflatoxin exposure *in utero* (ie by measuring biomarker levels in the pregnant women) was not performed.

For peer review only

Introduction

Aflatoxin (AF) is a mycotoxin produced mainly by *Aspergillus flavus* and *A. parasiticus* fungi that frequently contaminate crops in tropical and sub-tropical areas. It poses a great health risk to populations living in sub-Saharan Africa (SSA), especially in regions where groundnuts and/or maize are the staple foods.[1] Exposure to aflatoxin can begin in very early life; *in utero* through trans-placental exposure, during early infancy through breast feeding, and then increasing as children are weaned onto family foods, such as maize porridge or peanut sauces.[2] Earlier studies conducted in The Gambia that measured either food contamination and/or AF biomarkers, showed that these populations are at a high risk of AF exposure through food consumption.[3–7]

AF is a human liver carcinogen,[8] and has been associated with childhood growth faltering.[9,10] An inverse relationship between aflatoxin-albumin adduct (AF-alb) levels in pregnant women and growth of their infants was reported,[5] as well as between AF-alb and growth in infants below 2 years old.[7,10] While data from animal studies suggests that aflatoxin modulates the immune response at the level of innate cell functions, antibody production, lymphocyte activation and proliferation and regulation of cytokine/chemokine expression, there have been few studies in humans.[11,12] A reduction of salivary IgA expression,[13] and lower percentage of lymphocytes,[14,15] in children with high aflatoxin exposure have been reported. A greater understanding of the impact of chronic aflatoxin exposure during infancy and early childhood on immune system development is required.

The thymus is a primary lymphoid organ, essential for the development and differentiation of T lymphocytes. To investigate immune function of children, thymic index (a derived estimate of thymic volume) measured sonographically has been used as an indicator of immune development. Impaired thymic development in infancy has been associated with morbidity and mortality in childhood in Guinea Bissau and Bangladesh,[16–18] indicating a functional consequence of poor early thymic development.

The effect of chronic aflatoxin exposure on humoral immunity is less consistent in animal and human studies.[19,20] A study conducted in infants in the Gambia reported a weak but significant positive association between the level of AF-alb and pneumococcal antibody (Ab) titres.[13] Combined DTP (diphtheria, tetanus and pertussis) vaccine has been involved in the Expanded Programme on Immunization which was established in 1977 by the WHO and is estimated to prevent two to three million deaths in children every year.

For peer review only - http://bmjopen.bmj.com/site/about/guidelines.xhtml

The aim of the current study was to determine the impact of aflatoxin exposure in early infancy on thymus growth and altered antibody response to combined DTP vaccination.

Methods

Study subjects

The current sub-study into the effects of aflatoxin exposure on immune parameters was embedded within the Early Nutrition and Immune Development (ENID) trial (ISRCTN49285450). Full details of the ENID trial protocol have been published.[21] The primary objective of the ENID trial was to investigate whether combined pre-natal (protein-energy and/or micronutrients) and infant (micronutrient) nutritional supplements could improve child immune development. In total, 875 pregnant women from the West Kiang region of The Gambia were recruited into the ENID trial. In the current study, blood samples were taken from children born into the ENID trial between May 2011 and December 2012 (N=374), to assess aflatoxin exposure.

At enrolment into the ENID trial, pregnant women were randomly allocated to four different trial arms: Iron-folate (FeFol), multiple micronutrients (MMN), protein-energy (PE) combined with FeFol and PE combined with MMN. Supplementation continued until delivery. From 6 to 12 months of age, infants were further randomised into two supplementation arms; lipid-based nutritional supplementation (LNS) or LNS+MMN. Compliance of the supplementation was also recorded, through weekly interview.

For the current analysis, anthropometric measurements collected at 1, 8, 24 and 52 weeks of infant age and infant serum samples collected at 12, 24 and 52 weeks of age were used. Infant morbidity and feeding practices were recorded by field assistants who visited the children weekly at home. Morbidity questionnaires collected specific data on any episodes of diarrhoea, rapid breathing, vomiting, cough or fever or other symptoms during previous seven days. At the same weekly visit, data on infant feeding practices (breast feeding practices and introduction of non-breast milk foods) were also collected, using a standardised questionnaire.

Thymus size assessment

Thymus size of infants was assessed sonographically at 1, 8, 24 and 52 weeks of age using a validated method.[22] The transverse diameter of the thymus and the sagittal area of its largest lobe were detected and multiplied to give a volume-related thymic index (TI). Full details of the thymic index measurements of the ENID trial can be found in Moore *et al.* (2019).[23]

For peer review only - http://bmjopen.bmj.com/site/about/guidelines.xhtml

Antibody response to vaccination assessment

All infants were immunized in accordance with the Gambia EPI programme.[21] Three injections of combined DTP were given to children at week 8, 12 and 16. Venous blood samples collected from infants at 12 and 24 weeks and were used to determine the antibody response to vaccination. A multiple immunoassay based on Luminex xMAP technology was used to detect serum-specific IgG antibody responses against diphtheria toxoid, pertussis toxin and tetanus toxin.[24,25] Full details of the outcomes (antibody response to vaccination) of the ENID trial can be found in Okala *et al.* (2019).[26]

Aflatoxin exposure measurement

AF-alb levels in serum taken at 24 and 52 weeks of age were measured at the University of Leeds using a previously validated ELISA method.[27] In brief, this analysis is performed in four steps: albumin extraction and quantification, hydrolysis of albumin with pronase, purification of aflatoxin-albumin residues, and the competitive ELISA. The ELISA involves the pre-mixing of standards, samples, or controls with the rabbit anti aflatoxin-Cl_2-BSA polyclonal antibody, followed by competitive ELISA in which remaining unbound antibody can bind to aflatoxin ovalbumin conjugates coated on the surface of the ELISA plate well. After washing, the bound primary antibody is detected by incubation with an enzyme labelled goat anti-rabbit IgG secondary antibody. Control samples at four known concentrations of AF-alb were examined alongside each batch of samples.

Samples were measured at least twice, in triplicate each time, on two different days. Only results with a coefficient of variation (CV) less than 25% were accepted. The limit of detection (LOD) of the assay was 3 pg/mg albumin. The AF-alb level in samples less than LOD were assigned a value of 1.5 pg/mg for statistical analyses.

Statistical analysis

Statistical analysis was performed using STATA version 15 (StataCorp LP). Distribution of AF-alb level, TI and Ab response to vaccination data were skewed and were therefore log transformed prior to further analysis. Ab fold change was calculated as the Ab level at 24 weeks divided by Ab level at 12 weeks. Results were divided into low and high AF exposure groups at both 24 weeks and 52 weeks based on the median of AF-alb level at each time point. Student's *t-test* was used to compare the association of parameters in different seasons or low/high AF exposure level groups.

For peer review only - http://bmjopen.bmj.com/site/about/guidelines.xhtml

For other covariates, the season of sampling was categorised as rainy and dry season based on the date of TI measurement and blood sample collection for Ab and AF-alb analysis. There is a distinct seasonal pattern in The Gambia with a period of rainfall from July to October (rainy season) and a long dry season between November and June. The age (months) of introduction of non-breast milk foods to infants were recorded. Infant morbidity was coded as sum of the number of morbidity episodes (diarrhoea, vomiting, cough, rapid breathing and fever).

Relationships between AF-alb level and TI or Ab titre were explored using ordinary least squares regression for individual time points, or random effects model for the pooling data of all time points. Data are presented in different models for TI and Ab titres. The correlation between AF-alb level and TI were analysed as model 1: unadjusted; model 2: adjusted for infant size (length), sex and season at TI measurement; model 3: adjusted for infant size (length), season at TI measurement, sex and maternal supplement groups (for age <= 24 weeks) or both maternal and infants supplement groups (for age at 52 weeks). The correlation between AF-alb level and Ab response were analysed as model 1: unadjusted; model 2: adjusted for weight for height z-score (WHZ) and season of sample collection; model 3: adjusted for WHZ and season of sample collection, sex, maternal supplement groups, haemoglobin (Hb) levels and morbidity.

Ethical approval and consent to participate

Ethical approval for the ENID trial, and the aflatoxin sub-study was obtained from the joint Gambian Government/Medical Research Council (MRC) Unit The Gambia ethics committee (SCC1126v2, L2013.40). Written informed consent was obtained from all participants.

Results

A total of 800 live born infants were delivered into the main ENID trial.[23] For the current sub-study, 374 infants were included. Table 1 summarizes the characteristics of the participants in the current sub-study. More than half of mothers (67%) had less than one-year of formal education. Twenty-five out of 374 (6.7%) children were born with a low birth weight (<2500g). The mean (SD) duration of exclusive breast feeding was 5.2 (1.3) months.

For peer review only

Table 1. Characteristics of children in current study

Variable	n	Mean ± SD / n (%)
Gender, n (%)	374	
Male		192 (51.3)
Female		182 (48.7)
Ethnicity, n (%)	348	
Fula		30 (8.6)
Jola		11 (3.2)
Mandinka		304 (87.4)
Other		3 (0.9)
Mother's education, n (%)	352	
<1 year formal education		235 (66.8)
>1 year formal education		117 (33.2)
Birth weight (kg)	335	3.0 ± 0.4
Birth length (cm)	335	49.7 ± 2.0
Age of introduction of non-breast milk foods (weeks)		
<6 months	248	4.6 ± 1.3
>6 months	125	6.2 ± 0.1
Morbidity		
sum of first 12 weeks of life	360	8.3 ± 11.0
sum of first 24 weeks of life	361	18.7 ± 18.3
Infant supplementation group (6 months), n (%)	374	
LNS+MMN		192 (51.3)
LNS only		182 (48.7)
**AF-alb (pg/mg)**		**GM (95% CI)**
Week 24	352	3.52 (3.15, 3.94)
Week 52	331	25.39 (22.37, 28.82)

Aflatoxin exposure

The geometric mean (GM) level of AF-alb was 3.52 pg/mg at 24 weeks of age and increased to 25.39 pg/mg at 52 weeks with 52% and 2% of samples having levels lower than the LOD (3 pg/mg) at the 24 and 52 weeks age point, respectively. Seasonal variations were observed in the exposure (Figure 1) with around two-fold higher levels of AF-alb determined in samples collected during the dry season compared to the rainy season at both 24 and 52 weeks of age (GM 24wk: 2.5 vs 4.3 pg/mg, p<0.0001; 52wk: 19.3 vs 35.5 pg/mg, p<0.0001).

Thymic index

For peer review only

Thymus growth showed an upward trend during the first 24 weeks of age (Table 2). A significant increase was shown from birth to 8 weeks of age and reached maximum size at 24 weeks, followed by a decrease by 52 weeks. Seasonal variations were also identified in TI, with the TI values measured during the dry season being slightly higher than those measured in the rainy season at all time points, but this difference was only statistically significant in infants when measured at 8 weeks of age (mean 14.3 vs 13.3 cm^3, p=0.0136) (Figure 2).

Table 2. Mean level of thymic index and geometric mean level of antibody response to vaccination in the ENID trial and the current sub-study.

	n	Current sub-study GM (95%CI) / Mean ± SD / (%)[a]	n	ENID trial GM (95%CI) / Mean ± SD / (%)[a]
Thymic index (cm^3)				
Week 1	371	9.21 ± 3.13	765	9.18 ± 3.08
Week 8	369	13.97 ± 4.01	752	13.9 ± 4.09
Week 24	372	14.77 ± 4.25	747	14.7 ± 4.20
Week 52	362	13.59 ± 3.4	707	13.2 ± 3.71
Ab response to vaccination				
Pertussis (EU/ml)				
Week 12	355	7.61 (6.77, 8.57) / 69.3%	711	5.52 (5.04, 6.03) / 50.1%
Week 24	322	132.37 (111.44, 157.22) / 96.0%	663	89.81 (77.71, 103.79) / 88.2%
Diphtheria (IU/ml)				
Week 12	355	0.26 (0.22, 0.32) / 72.4 %	711	0.12 (0.11, 0.14) / 55.5%
Week 24	322	1.18 (1.04, 1.34) / 94.1%	663	1.40 (1.30, 1.51) / 96.8%
Tetanus (IU/ml)				
Week 12	355	0.83 (0.75, 0.91) / 98.9%	711	0.64 (0.59, 0.68) / 97.3%
Week 24	322	4.34 (3.81, 4.94) / 99.7%	663	3.71 (3.40, 4.05) / 99.6%
Morbidity				
Sum of 12 weeks	360	8.3 ± 10.9	729	9.6 ± 12.1
Sum of 24 weeks	361	18.7 ± 18.3	730	22.8 ± 23.5

International standards protective antibody titres (WHO): diphtheria and tetanus >0.1 IU/ml;[24,28] pertussis >5.0 EU/ml.[29] The (%)[a] presented the percentage of samples had protective antibody titres.

Antibody response to DTP vaccination

The Ab response to vaccination increased significantly at 24 weeks of age compared to 12 weeks (Table 2). For pertussis, the protective Ab titres were detected in 69.3% of infants after the first dose, increasing to 96.0% at 24 weeks. For diphtheria, 72.4% of infants at 12 weeks and 94.1% at 24 weeks showed protective levels. For tetanus, the rates were 98.9% and 99.7%

For peer review only - http://bmjopen.bmj.com/site/about/guidelines.xhtml

between the two time points. Ab titres were slightly higher in samples collected during the rainy season at 12 weeks of age, but lower at 24 weeks of age (Figure 3).

Primary outcomes of ENID trial vs ENID-sub study

Table 2 compares the mean TI value and GM level of Ab titres in our aflatoxin sub-study and the full ENID trial. Infants included in our sub-study showed no significant difference in mean TI when compared with the overall 800 infants in the ENID trial. In terms of the Ab response titres, except for the Ab response to diphtheria at 24 weeks, both the GM of Ab titres and the percentage of samples with protective antibody titres were higher in the sub-group studied here than in the full cohort of ENID infants. However, morbidity was higher among the ENID trial subjects than in our sub-samples at both 12 and 24 weeks.

Aflatoxin exposure and primary outcomes

Figures 2 and 3 present the TI and Ab titre in the low and high AF-alb groups. The mean TI was lower in the high AF-alb group than in the low AF-alb group during the first 24 weeks, but the difference was only statistically significant at 8 weeks (mean 14.9 vs 13.1 cm^3, $p<0.0001$), and not present at 52 weeks (Figure 2). There were no significant differences for Ab response to pertussis and tetanus vaccines between the low and high AF-alb groups (Figure 3). However, a significantly higher Ab titre against diphtheria was determined in infants in the high AF-alb group compared to those in the low AF-alb group at both time points (Ab GM at wk12 : 0.16 vs 0.46 IU/ml $p<0.0001$; Ab GM at wk24 : 0.96 vs 1.46 IU/ml, $p=0.0011$).

Regression analysis with or without adjusting for the other variables showed there were no significant associations between AF-alb level and TI at 1, 24 or 52 weeks (Table 3). However, significant negative correlations were determined between AF-alb and TI at 8 weeks in all unadjusted and adjusted models. In addition, the random effect model, which measured the AF-alb correlation with all time points of TI pooled data also showed significantly negative correlations at 24 weeks in both unadjusted and adjusted models, and at 52 weeks in adjusted models.

For peer review only - http://bmjopen.bmj.com/site/about/guidelines.xhtml

Table 3. Relationship between aflatoxin-albumin level and thymic index

	Model 1		Model 2		Model 3	
	Coef. (SE)	p	Coef. (SE)	p	Coef. (SE)	p
Week 1	-0.0083 (0.018)	0.637	-0.0012 (0.018)	0.95	-0.0012 (0.018)	0.948
Week 8	-0.0606 (0.014)	<0.001	-0.056 (0.014)	<0.001	-0.056 (0.014)	<0.001
Week 24	-0.0098 (0.014)	0.49	-0.015 (0.015)	0.3	-0.015 (0.015)	0.301
Week 52	-0.0115 (0.014)	0.399	-0.014 (0.014)	0.296	-0.014 (0.014)	0.301
all time points to infant age <24 weeks	-0.027 (0.011)	0.014	-0.023 (0.010)	0.022	-0.023 (0.010)	0.021
all time points to infant age <52 weeks	0.0076 (0.007)	0.28	-0.053 (0.007)	<0.001	-0.052 (0.007)	<0.001

Notes:
TI at 1, 8 and 24 weeks were analysed with AF-alb level at 24 weeks. TI at 52 weeks was analysed with AF-alb level at 52 weeks.
Model 1 unadjusted
Model 2 adjusted for infant size (length), sex and season at TI measurement
Model 3 adjusted for infant size (length), season at TI measurement, sex and maternal supplement groups (for age <= 24 weeks) & infants supplement groups (for age at 52 weeks)

Regression analysis confirmed a significant positive correlation between AF exposure level and Ab response to diphtheria at both 12 and 24 weeks of age (p<0.001 & p=0.002, respectively), but there were no significant associations between AF-alb level and Ab response to pertussis and tetanus (Table 4).

Neither maternal nor infant supplementation had a significant effect on AF-alb level, TI or Ab titre at any time point (data not presented).

For peer review only

Table 4. Relationship between aflatoxin-albumin level and antibody response to vaccination

	Model 1		Model 2		Model 3	
	**Coef. (SE)**	**p**	**Coef. (SE)**	**p**	**Coef. (SE)**	**p**
**Pertussis**						
Week 12	-0.093 (0.058)	0.11	-0.093 (0.060)	0.123	-0.088 (0.070)	0.207
Week 24	0.143 (0.083)	0.085	0.133 (0.086)	0.124	0.099 (0.096)	0.301
Fold change	0.190 (0.109)	0.083	0.197 (0.113)	0.084	0.127 (0.127)	0.319
**Diphtheria**						
Week 12	0.421 (0.093)	<0.001	0.418 (0.094)	<0.001	0.495 (0.109)	<0.001
Week 24	0.186 (0.058)	0.002	0.190 (0.061)	0.002	0.204 (0.064)	0.002
Fold change	-0.239 (0.097)	0.014	-0.124 (0.097)	0.204	-0.149 (0.106)	0.162
**Tetanus**						
Week 12	0.065 (0.048)	0.175	0.065 (0.049)	0.182	0.096 (0.056)	0.089
Week 24	-0.034 (0.062)	0.578	-0.064 (0.064)	0.322	-0.071 (0.070)	0.316
Fold change	-0.111 (0.081)	0.172	-0.096 (0.084)	0.252	-0.127 (0.092)	0.168

Notes:
Antibody response to vaccination were analysed with AF-alb level at 24 weeks only.
Model 1 unadjusted
Model 2 adjusted for WHZ and season of sample collection
Model 3 adjusted for WHZ and season of sample collection, sex, maternal supplement group, Hb levels and morbidity.

Discussion

This is the first study to investigate the impact of aflatoxin exposure on immune function of infants less than 12 months of age using TI and antibody response to DTP vaccination as indicators of immune function. Infants who were exposed to higher levels of aflatoxin, had smaller thymus during the first 24 weeks of age, and had significantly higher protective Ab titres against diphtheria.

The observed high prevalence of AF exposure in the present study is consistent with previous findings in the same region of Gambia.[5,30,31] An increasing prevalence and upward trend of AF-alb levels with age were determined in our study. Infants at 52 weeks showed more than seven-fold higher levels of AF-alb than at 24 weeks of age. Aflatoxin exposure could happen *in utero* through trans-placental exposure, and the exposure level will increase during the first year when infants are gradually introduced to complementary foods.[2]

For peer review only - http://bmjopen.bmj.com/site/about/guidelines.xhtml

The Gambia has pronounced dry and rainy seasons, and previous studies have shown a seasonal impact on both growth and aflatoxin exposure.[6,32] In the present study, serum samples collected during the dry season had significantly higher levels of AF-alb than those samples collected during the rainy season. The annual dry season in The Gambia is a time of relative food availability, as food supplies from the previous harvest are usually plentiful. Aflatoxin contamination in foods and crops tends to increase after a period of storage.[33] Populations are more likely to consume old grains towards the end of the dry (harvest) season, and foods which have been stored for a period are more likely to be contaminated. Our findings are consistent with previous studies in Benin and Guinea.[10,34] However, exposure can also depend on food type. In Senegal, Watson *et al.* (2015) found that higher AF-alb levels in the harvest season compared to the post-harvest season correlated with the recorded high consumption (four or more days a week) of contaminated groundnuts during the harvest period, with groundnuts being more susceptible to contamination than maize.[35]

Similar growth trend and seasonal variation of TI were previously reported in Gambia by Collinson *et al.* (2003) who also reported the consistently smaller thymus size in the rainy season, and significant difference at 8 weeks of age ($p=0.001$).[36] The significant retardation of thymus at 8 weeks found in children with high aflatoxin exposure might due to it is the time point that the thymus grows fastest. The sonographic method used in the current study to assess TI only represents an anatomical feature and not function, but previous studies in animals and infants have demonstrated a correlation between thymus size and lymphocyte proportion and function [37,38]. Therefore, smaller TI in infants could predict a lower immunity in the future. Previously, AF induced damage on the thymus has only been investigated in animal models. A recent study reported that AF caused thymic histopathological lesions and pathological impairments in chickens which had been fed with aflatoxin contaminated feeds (34.3–134 μg AFB_1/kg corn feed) for 21 and 42 days.[38] The reduction in thymus size and number of apoptotic lymphocytes occurred in a dose-dependent manner.

There was no previous study investigating the influence of aflatoxin exposure on Ab response to DTP vaccination. In our study, we found a significant positive association between aflatoxin biomarker level and Ab titre to diphtheria. An earlier study conducted in Gambian infants determined a similar weak but significant positive correlation between AF-alb level and one serotype of anti-pneumococcal Ab.[13] An animal study to induce anti-aflatoxin B_1 antibody in dairy cows which combined AFB_1 with recombinant diphtheria toxin molecules injected into heifers boosted the generation of anti-AFB_1 Abs.[39] The effect of AF on Ab response could vary depending on the feature of the vaccines, as early animal studies conducted in chicken

For peer review only - http://bmjopen.bmj.com/site/about/guidelines.xhtml

and rabbits reported inverse influence of AF exposure level on Ab response to different vaccines.[40,41] The mechanism behind the significant positive correlation between AF-alb level and Ab titre to diphtheria needs further investigation.

One limitation of the current study was the AF-alb levels were not measured at the birth of infants or in the maternal blood. However, the AF-alb biomarker integrates exposure over the previous 2-3 months,[42] therefore measuring AF-alb at 24 weeks is a good proxy for aflatoxin exposure in early infancy. Turner *et al.* (2007) reported a close association of AF-alb levels in maternal and infants' cord blood, and also reported a significant negative correlation between maternal AF biomarker level and child weight and height gain at the first year of life.[5] In our study, we found the TI was significantly correlated with the weight of infants in the first year. Considering the AF associated growth retardation determined in infants in the current cohort,[7] we suggest that AF could be a potential factor that influenced child growth and thymus development during the early stages of life.

A few previous studies have examined the effect of high levels of aflatoxin exposure on markers of immunity in human. Turner *et al.* (2003) reported a decrease in expression of salivary IgA in children from rural Gambia with detectable AF-alb levels compared with those with undetectable levels.[13] A lower percentage of T and B cells have been observed in participants with high concentrations of AF-alb in Ghanaians aged between 19 and 86 years old.[14,15] However, some animal studies of aflatoxin induced effects on the immune system are inconsistent: Li *et al.* (2014) and Meissonnier *et al.* (2008) found no significant effect of aflatoxin B_1 on humoral immunity function in chickens and pigs.[43,44] While another study found increased expression of IgM and IgG in pigs dosed with high AF contaminated feed.[45] The mechanism of the immunoglobulin rise is unclear.

In conclusion, we have demonstrated a significant effect of aflatoxin exposure on infant immune development, assessed by thymic size. Less consistent evidence was found for infants' antibody response to vaccination. This study adds to evidence that aflatoxin exposure in infants can modify the immune response, and further efforts should be made to ameliorate dietary aflatoxin exposure across populations in sub-Saharan Africa.

Acknowledgements

The research reported here was funded by the Meridian Institute (Agreement Number 9678.0) and the Bill and Melinda Gates Foundation (grant no: OPP1066947). The ENID trial was

For peer review only - http://bmjopen.bmj.com/site/about/guidelines.xhtml

supported by the UK Medical Research Council (MRC) (MC-A760-5QX00) and the UK Department for International Development (DFID) under the MRC/ DFID Concordat agreement. The dataset analysed in this paper is available from the corresponding author on reasonable request, and with appropriate additional ethical approvals, where necessary.

Contributors

MNR, YYG, AMP and SEM contributed to the original study design and implementation. YX, GC, PN and TF have contributed to laboratory analysis. YX wrote the first draft of the manuscript. All authors approved the final version of the manuscript.

Patient and public involvement

Patients or the public were not involved in the design, conduct, reporting or dissemination plans of our research.

Data sharing statement

Data not included in the manuscript are available upon reasonable request

References

1 Gong YY, Watson S, Routledge MN. Aflatoxin Exposure and Associated Human Health Effects, a Review of Epidemiological Studies. *Food Saf* 2016;4:14–27. doi:10.14252/foodsafetyfscj.2015026

2 Gong YY, Egal S, Hounsa A, *et al.* Determinants of aflatoxin exposure in young children from Benin and Togo, West Africa: The critical role of weaning. *Int J Epidemiol* 2003;32:556–62. doi:10.1093/ije/dyg109

3 Hudson GJ, Wild CP, Zarba A, *et al.* Aflatoxins isolated by immunoaffinity chromatography from foods consumed in the Gambia, West Africa. *Nat Toxins* 1992;1:100–5. doi:10.1002/nt.2620010208

4 Wild P, Whittle C, Hall J, *et al.* Liver Enzymes , and Hepatitis Gambian B Virus Infection in. 1993;2:555–62.

5 Turner PC, Collinson AC, Cheung YB, *et al.* Aflatoxin exposure in utero causes growth faltering in Gambian infants. *Int J Epidemiol* 2007;36:1119–25. doi:10.1093/ije/dym122

For peer review only - http://bmjopen.bmj.com/site/about/guidelines.xhtml

6 Castelino JM, Dominguez-Salas P, Routledge MN, *et al.* Seasonal and gestation stage associated differences in aflatoxin exposure in pregnant Gambian women. *Trop Med Int Heal* 2014;19:348–54. doi:10.1111/tmi.12250

7 Watson S, Moore SE, Darboe MK, *et al.* Impaired growth in rural Gambian infants exposed to aflatoxin: A prospective cohort study. *BMC Public Health* 2018;18 doi:10.1186/s12889-018-6164-4

8 IARC. Some traditional herbal medicines, some mycotoxins, naphthalene and styrene. *IARC Monogr Eval Carcinog Risks Hum* 2002;82:1–556. doi:10.1002/food.19940380335

9 Gong YY, Cardwell K, Hounsa A, *et al.* Dietary aflatoxin exposure and impaired growth in young children from Benin and Togo: Cross sectional study. *Br Med J* 2002;325:20–1. doi:10.1136/bmj.325.7354.20

10 Gong Y, Hounsa A, Egal S, *et al.* Postweaning exposure to aflatoxin results in impaired child growth: A longitudinal study in Benin, West Africa. *Environ Health Perspect* 2004;112:1334–8. doi:10.1289/ehp.6954

11 Bbosa GS, Kitya D, Lubega A, *et al.* Review of the Biological and Health Effects of Aflatoxins on Body Organs and Body Systems. In: *Aflatoxins - Recent Advances and Future Prospects*. 2013. doi:10.5772/51201

12 Wild C, Miller J, Groopman J. *Mycotoxin Control in Low- and Middle- Income Countries*. 2015. http://www.ncbi.nlm.nih.gov/pubmed/27030861

13 Turner PC, Moore SE, Hall AJ, *et al.* Modification of immune function through exposure to dietary aflatoxin in Gambian children. *Environ Health Perspect* 2003;111:217–20. doi:10.1289/ehp.5753

14 Jiang Y, Jolly PE, Ellis WO, *et al.* Aflatoxin B1 albumin adduct levels and cellular immune status in Ghanaians. *Int Immunol* 2005;17:807–14. doi:10.1093/intimm/dxh262

15 Jiang Y, Jolly PE, Preko P, *et al.* Aflatoxin-related immune dysfunction in health and in human immunodeficiency virus disease. *Clin Dev Immunol* 2008;790309. doi:10.1155/2008/790309

16 Aaby P, Marx C, Trautner S, *et al.* Thymus size at birth is associated with infant mortality: A community study from Guinea-Bissau. *Acta Paediatr Int J Paediatr* 2002;91:698–703. doi:10.1080/080352502760069142

17 Garly ML, Trautner SL, Marx C, *et al.* Thymus Size at 6 Months of Age and Subsequent Child

For peer review only

Mortality. *J Pediatr* 2008;153. doi:10.1016/j.jpeds.2008.04.069

18 Moore SE, Fulford AJC, Wagatsuma Y, *et al.* Thymus development and infant and child mortality in rural Bangladesh. *Int J Epidemiol* 2014;43:216–23. doi:10.1093/ije/dyt232

19 Bondy GS, Pestka JJ. Immunomodulation by fungal toxins. *J Toxicol Environ Heal - Part B Crit Rev* Published Online First: 2000. doi:10.1080/109374000281113

20 Meissonnier GM, Marin DE, Galtier P, *et al.* Modulation of the immune response by a group of fungal food contaminant, the aflatoxins. *Nutr Immun* 2006.

21 Moore SE, Fulford AJC, Darboe MK, *et al.* A randomized trial to investigate the effects of pre-natal and infant nutritional supplementation on infant immune development in rural Gambia: The ENID trial: Early Nutrition and Immune Development. *BMC Pregnancy Childbirth* 2012;12:1–8. doi:10.1186/1471-2393-12-107

22 Hasselbalch H, Nielsen MB, Jeppesen D, *et al.* Sonographic measurement of the thymus in infants. *Eur Radiol* 1996;6:700–3. doi:10.1007/BF00187675

23 Moore SE, Fulford AJC, Sosseh F, *et al.* Thymic size is increased by infancy, but not pregnancy, nutritional supplementation in rural Gambian children: A randomized clinical trial. *BMC Med* 2019;17:1–9. doi:10.1186/s12916-019-1264-2

24 Colombet I, Saguez C, Sanson-Le Pors MJ, *et al.* Diagnosis of tetanus immunization status: Multicenter assessment of a rapid biological test. *Clin Diagn Lab Immunol* 2005;12:1057–62. doi:10.1128/CDLI.12.9.1057-1062.2005

25 van Gageldonk PGM, van Schaijk FG, van der Klis FR, *et al.* Development and validation of a multiplex immunoassay for the simultaneous determination of serum antibodies to Bordetella pertussis, diphtheria and tetanus. *J Immunol Methods* Published Online First: 2008. doi:10.1016/j.jim.2008.02.018

26 Okala SG, Darboe MK, Sosseh F, *et al.* Impact of nutritional supplementation during pregnancy on antibody responses to diphtheria-tetanus-pertussis vaccination in infants: A randomised trial in the Gambia. *PLoS Med* 2019;16. doi:10.1371/journal.pmed.1002854

27 Chapot B, Wild C. *ELISA for quantification of aflatoxin-albumin adducts and their application to human exposure assessment*. Academic Press, San Diego 1991.

28 Efstratiou A, Maple PC. Manual for the laboratory diagnosis of diphtheria. Expand. Program. Immun. Eur. Reg. WHO. 1994.

For peer review only - http://bmjopen.bmj.com/site/about/guidelines.xhtml

29 van Gageldonk PGM, van Schaijk FG, van der Klis FR, *et al.* Development and validation of a multiplex immunoassay for the simultaneous determination of serum antibodies to Bordetella pertussis, diphtheria and tetanus. *J Immunol Methods* 2008;335:79–89. doi:10.1016/j.jim.2008.02.018

30 Turner PC, Mendy M, Whittle H, *et al.* Hepatitis B infection and aflatoxin biomarker levels in Gambian children. *Trop Med Int Heal* 2000;5:837–41. doi:10.1046/j.1365-3156.2000.00664.x

31 Wild CP, Yin F, Turner PC, *et al.* Environmental and genetic determinants of aflatoxin-albumin adducts in The Gambia. *Int J Cancer* 2000;86:1–7. doi:10.1002/(SICI)1097-0215(20000401)86:1<1::AID-IJC1>3.0.CO;2-I

32 Nabwera HM, Fulford AJ, Moore SE, *et al.* Growth faltering in rural Gambian children after four decades of interventions: a retrospective cohort study. *Lancet Glob Heal* 2017;5:e208–16. doi:10.1016/S2214-109X(16)30355-2

33 Pitt JI, Taniwaki MH, Cole MB. Mycotoxin production in major crops as influenced by growing, harvesting, storage and processing, with emphasis on the achievement of Food Safety Objectives. *Food Control* 2013;32:205–15. doi:10.1016/j.foodcont.2012.11.023

34 Turner PC, Sylla A, Gong YY, *et al.* Reduction in exposure to carcinogenic aflatoxins by postharvest intervention measures in west Africa: A community-based intervention study. *Lancet* 2005;365:1950–6. doi:10.1016/S0140-6736(05)66661-5

35 Watson S, Diedhiou PM, Atehnkeng J, *et al.* Seasonal and geographical differences in aflatoxin exposures in Senegal. *World Mycotoxin J* 2015;8:525–31. doi:10.3920/WMJ2014.1824

36 Collinson AC, Moore SE, Cole TJ, *et al.* Birth season and environmental influences on patterns of thymic growth in rural Gambian infants. *Acta Paediatr Int J Paediatr* 2003;92:1014–20. doi:10.1080/08035250310004379

37 Jeppesen DL, Hasselbalch H, Lisse IM, *et al.* T-lymphocyte subsets, thymic size and breastfeeding in infancy. *Pediatr Allergy Immunol* 2004;15:127–32. doi:10.1111/j.1399-3038.2004.00032.x

38 Peng X, Bai S, Ding X, *et al.* Pathological impairment, cell cycle arrest and apoptosis of thymus and bursa of fabricius induced by aflatoxin-contaminated corn in broilers. *Int J Environ Res Public Health* 2017;14. doi:10.3390/ijerph14010077

39 Giovati L, Gallo A, Masoero F, *et al.* Vaccination of heifers with anaflatoxin improves the reduction of aflatoxin B1 carry over in milk of lactating dairy cows. *PLoS One* 2014;9.

For peer review only - http://bmjopen.bmj.com/site/about/guidelines.xhtml

doi:10.1371/journal.pone.0094440

40 Azzam AH, Gabal MA. Interaction of aflatoxin in the feed and immunization against selected infectious diseases. I. Infectious bursal disease. *Avian Pathol* 1997;26:317–25. doi:10.1080/03079459708419214

41 Dimitri RA, Gabal MA. Immunosuppressant activity of aflatoxin ingestion in rabbits measured by response to mycobacterium bovis antigen I. Cell mediated immune response measured by skin test reaction. *Vet Hum Toxicol* 1996;38:333–6.

42 Wild CP, Hasegawa R, Barraud L, *et al.* Aflatoxin-albumin adducts: a basis for comparative carcinogenesis between animals and humans. *Cancer Epidemiol Biomarkers Prev* 1996;5:179–89.http://www.ncbi.nlm.nih.gov/pubmed/8833618

43 Li Y, Ma QG, Zhao LH, *et al.* Effects of lipoic acid on immune function, the antioxidant defense system, and inflammation-related genes expression of broiler chickens fed aflatoxin contaminated diets. *Int J Mol Sci* 2014;15:5649–62. doi:10.3390/ijms15045649

44 Meissonnier GM, Pinton P, Laffitte J, *et al.* Immunotoxicity of aflatoxin B1: Impairment of the cell-mediated response to vaccine antigen and modulation of cytokine expression. *Toxicol Appl Pharmacol* 2008;231:142–9. doi:10.1016/j.taap.2008.04.004

45 Panangala VS, Giambrone JJ, Diener UL, *et al.* Effects of aflatoxin on the growth performance and immune responses of weanling swine. *Am J Vet Res* 1986;47:2062–7.

For peer review only

Figure Legends

Figure 1. Aflatoxin-albumin level (log transformed) in rainy and dry season at 24 and 52 weeks.

Figure 2. Thymic index in children in low or high Aflatoxin-albumin group or in different seasons. The low and high AF-alb group was divided by the median level at each time point. TI at week 8, 12 and 24 were group by AF-alb at week 24, TI at 52 weeks was group by AF-alb level at week 52. The season of TI was defined as the time of measurement conducted.

Figure 3. Antibody level to vaccination in low or high aflatoxin-albumin group and in different seasons. The low and high AF-alb group was divided by the median level at

For peer review only - http://bmjopen.bmj.com/site/about/guidelines.xhtml

week 24. The season of Ab level was defined as the date of blood sample collection.

For peer review only

For peer review only - http://bmjopen.bmj.com/site/about/guidelines.xhtml

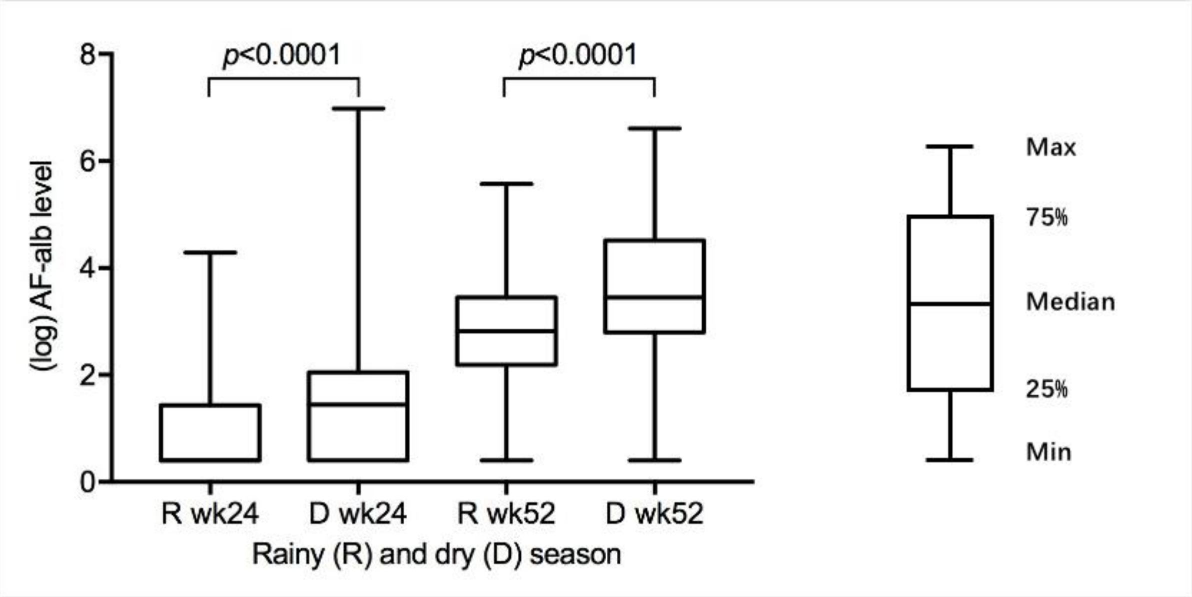

For peer review only

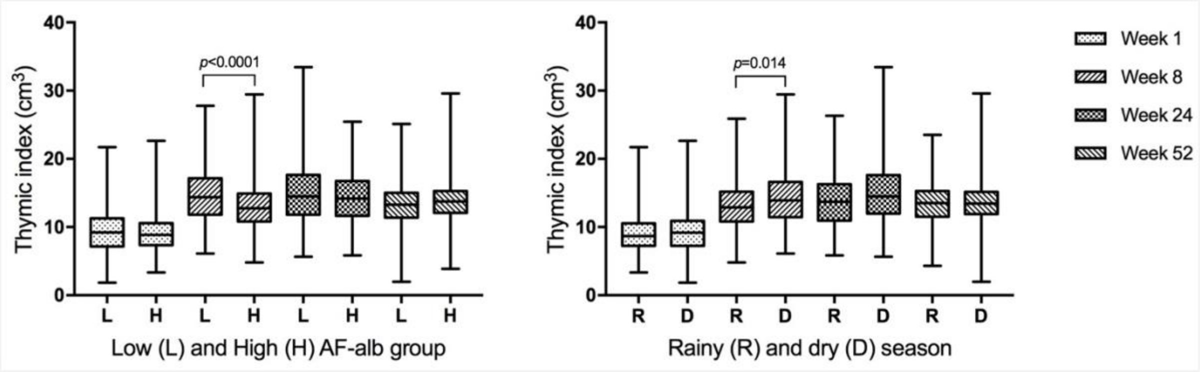
Thymic index (cm³)
p<0.0001
L
H
Low (L) and High (H) AF-alb group
p=0.014
R
D
Rainy (R) and dry (D) season
Week 1
Week 8
Week 24
Week 52

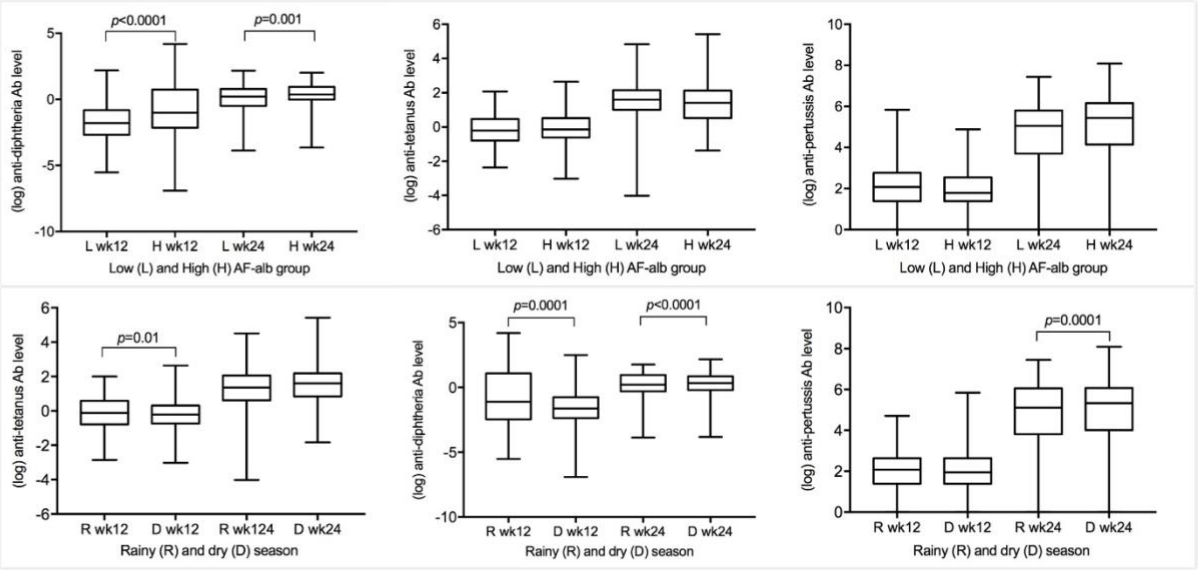

For peer review only

BMJ Open

Impact of dietary aflatoxin on immune development in Gambian infants: a cohort study

Journal:	*BMJ Open*
Manuscript ID	bmjopen-2021-048688.R1
Article Type:	Original research
Date Submitted by the Author:	25-May-2021
Complete List of Authors:	Xu, Ya; University of Leeds<br>Moore, Sophie; King's College London, Women and Children's Health<br>Chen, Gaoyun; Queen’s University Belfast<br>Nshe, Patrick; MRC Unit The Gambia at LSHTM<br>Faye-Joof, Tisbeh; MRC Unit The Gambia at LSHTM<br>Prentice, Andrew; MRC Unit The Gambia at LSHTM<br>Gong, Yun Yun; University of Leeds<br>Routledge, Michael; University of Leeds, School of Medicine; Jiangsu University, School of Food and Biological Engineering
**Primary Subject Heading**:	Epidemiology
Secondary Subject Heading:	Global health
Keywords:	Epidemiology < TROPICAL MEDICINE, Nutrition < TROPICAL MEDICINE, IMMUNOLOGY

SCHOLARONE™ Manuscripts

I, the Submitting Author has the right to grant and does grant on behalf of all authors of the Work (as defined in the below author licence), an exclusive licence and/or a non-exclusive licence for contributions from authors who are: i) UK Crown employees; ii) where BMJ has agreed a CC-BY licence shall apply, and/or iii) in accordance with the terms applicable for US Federal Government officers or employees acting as part of their official duties; on a worldwide, perpetual, irrevocable, royalty-free basis to BMJ Publishing Group Ltd ("BMJ") its licensees and where the relevant Journal is co-owned by BMJ to the co-owners of the Journal, to publish the Work in this journal and any other BMJ products and to exploit all rights, as set out in our licence.

The Submitting Author accepts and understands that any supply made under these terms is made by BMJ to the Submitting Author unless you are acting as an employee on behalf of your employer or a postgraduate student of an affiliated institution which is paying any applicable article publishing charge ("APC") for Open Access articles. Where the Submitting Author wishes to make the Work available on an Open Access basis (and intends to pay the relevant APC), the terms of reuse of such Open Access shall be governed by a Creative Commons licence – details of these licences and which Creative Commons licence will apply to this Work are set out in our licence referred to above.

Other than as permitted in any relevant BMJ Author's Self Archiving Policies, I confirm this Work has not been accepted for publication elsewhere, is not being considered for publication elsewhere and does not duplicate material already published. I confirm all authors consent to publication of this Work and authorise the granting of this licence.

For peer review only

Impact of dietary aflatoxin on immune development in Gambian infants: a cohort study

Ya Xu[1], Sophie E. Moore[2, 3], Gaoyun Chen[4], Patrick Nshe[3], Tisbeh Faye-Joof[3], Andrew M Prentice[3], Yun Yun Gong[5], Michael N Routledge[1,6*]

[1]School of Medicine, University of Leeds, Leeds LS2 9JT, UK,

[2]Department of Women and Children's Health, King's College London, St Thomas' Hospital, 10th Floor North Wing, London SE1 7EH, UK,

[3]MRC Unit The Gambia at the London School of Hygiene and Tropical Medicine, Atlantic Boulevard, Fajara, PO Box 273, Banjul, The Gambia,

[4]Institute for Global Food Security, School of Biological Sciences, Queen's University Belfast, Belfast, UK,

[5]School of Food Science and Nutrition, University of Leeds, Leeds, UK.

[6]School of Food and Biological Engineering, Jiangsu University, Zhenjiang, Jiangsu Province, China

*Correspondence: medmnr@leeds.ac.uk

For peer review only

Abstract

Background Chronic aflatoxin (AF) exposure has been shown to occur at high levels in children from sub-Saharan Africa (SSA), and has been associated with growth retardation and immune dysfunction. Our objective was to investigate the impact of aflatoxin exposure on immune development in early infancy using thymic size and antibody (Ab) response to vaccination as indicators of immune function.

Methods A total of 374 infants born between May 2011 and December 2012 were enrolled into the current study. These infants were recruited from a larger, randomized trial examining the impact of nutritional supplementation of mothers and infants on infant immune development (the ENID Trial; ISRCTN49285450). Thymic size (Thymic Index; TI) was measured by sonography at 1, 8, 24 and 52 weeks of infant age. Infants were given the diphtheria-tetanus-pertussis (DTP) vaccine at 8, 12 and 16 weeks of age, and antibody responses to each vaccine measured at 12 and 24 weeks of age. Aflatoxin-albumin (AF-alb) adduct levels in infant blood were measured by ELISA as the biomarker of aflatoxin exposure.

Results The geometric mean (GM) level of AF-alb increased with age. Only half of infants had detectable AF-alb with a GM of 3.52 pg/mg at 24 weeks, increasing to 25.39 pg/mg at 52 weeks, when 98% of infants had AF-alb >limit of detection. Significant negative association of AF-alb level with TI were seen in infants during the first 24 weeks, especially at 8 weeks of age ($p<0.001$), which is the time point of fastest thymus growth. There were no associations

For peer review only - http://bmjopen.bmj.com/site/about/guidelines.xhtml

between aflatoxin exposure level and antibody response to pertussis and tetanus, but a significant positive correlation was observed between AF-alb level and antibody titre to diphtheria ($p<0.005$).

Conclusions High levels of aflatoxin exposure during early infancy may impact on infant immune development.

Keywords Aflatoxin, immune development, thymus, antibody response to vaccination

Strengths and limitations of this study

- The study was embedded within a clinical trial (ENID), which reduced selection bias of subjects
- The subjects were part of a cohort naturally exposed to aflatoxin through their diet, which allowed for multiple time point sampling
- The aflatoxin exposure was measured using a validated biomarker, which allows better estimation of exposure compared to food intake estimates
- A limitation of the study is that aflatoxin exposure *in utero* (ie by measuring biomarker levels in the pregnant women) was not performed.

For peer review only

Introduction

Aflatoxin (AF) is a mycotoxin produced mainly by *Aspergillus flavus* and *A. parasiticus* fungi that frequently contaminate crops in tropical and sub-tropical areas. It poses a great health risk to populations living in sub-Saharan Africa (SSA), especially in regions where groundnuts and/or maize are the staple foods.[1] Exposure to aflatoxin can begin *in utero* through trans-placental exposure, continue in early infancy through breast feeding, and increase as children are weaned onto family foods, such as maize porridge or peanut sauces.[2] Populations in The Gambia are at a high risk of AF exposure through food consumption.[3–7]

AF is a human liver carcinogen,[8] and has also been associated with childhood growth faltering.[9,10] An inverse relationship has been reported between aflatoxin-albumin adduct (AF-alb) levels in pregnant women and subsequent infant growth [5] as well as between AF-alb and growth in infants below 2 years old.[7,10], and high levels of AF-lysine were associated with stunting and severe malnutrition in children in Nigeria, [11] and with underweight children in Kenya.[12] Data from animal studies suggests that aflatoxin modulates the immune response at the level of innate cell functions, antibody production, lymphocyte activation and proliferation and regulation of cytokine/chemokine expression, but there have been few studies on the impact of aflatoxin on immune function in humans.[13,14] A reduction of salivary IgA expression,[15] and lower percentage of lymphocytes,[16,17] in children with high aflatoxin exposure have been reported. A greater understanding of the impact of chronic aflatoxin exposure during infancy and early childhood on immune system development is required.

The thymus is a primary lymphoid organ, essential for the development and differentiation of T lymphocytes. Thymic index (a derived estimate of thymic volume) measured sonographically has been used as an indicator of immune development in infants. Impaired thymic development in infancy has been associated with morbidity and mortality in childhood in Guinea Bissau and Bangladesh,[18–20] indicating a functional consequence of poor early thymic development.

The effect of chronic aflatoxin exposure on humoral immunity is less consistent in animal and human studies.[21,22] A study conducted in infants in the Gambia reported a weak but significant positive association between the level of AF-alb and pneumococcal antibody (Ab) titres.[15] Combined DTP (diphtheria, tetanus and pertussis) vaccine has been involved in the Expanded Program on Immunization which was established in 1977 by the WHO and is estimated to prevent two to three million deaths in children every year.

For peer review only

The aim of the current study was to determine the impact of aflatoxin exposure in early infancy on thymus growth and altered antibody response to combined DTP vaccination.

Methods

Study subjects

The current sub-study into the effects of aflatoxin exposure on immune parameters was embedded within the Early Nutrition and Immune Development (ENID) trial (ISRCTN49285450). Full details of the ENID trial protocol have been published.[23] The primary objective of the ENID trial was to investigate whether combined pre-natal (protein-energy and/or micronutrients) and infant (micronutrient) nutritional supplements could improve child immune development. In total, 875 pregnant women from the West Kiang region of The Gambia were recruited into the ENID trial. In the current study, blood samples were taken from all children born into the ENID trial between May 2011 and December 2012 (N=374), to give sufficient power to assess impact of aflatoxin exposure on TI and antibody response.

At enrolment into the ENID trial, pregnant women were randomly allocated to four different trial arms: Iron-folate (FeFol), multiple micronutrients (MMN), protein-energy (PE) combined with FeFol and PE combined with MMN. Supplementation continued until delivery. From 6 to 12 months of age, infants were further randomised into two supplementation arms; lipid-based nutritional supplementation (LNS) or LNS+MMN. Compliance of the supplementation was also recorded, through weekly interview. Due to the rolling randomization into supplementation groups on enrolment, the children from whom samples were used in the current study were randomized into the various groups at approximately equal numbers (Table 1).

For the current analysis, anthropometric measurements collected at 1, 8, 24 and 52 weeks of infant age and infant serum samples collected at 12, 24 and 52 weeks of age were used. Infant morbidity and feeding practices were recorded by field assistants who visited the children weekly at home. Morbidity questionnaires collected specific data on any episodes of diarrhoea, rapid breathing, vomiting, cough or fever or other symptoms during previous seven days. At the same weekly visit, data on infant feeding practices (breast feeding practices and introduction of non-breast milk foods) were also collected, using a standardised questionnaire.

For peer review only - http://bmjopen.bmj.com/site/about/guidelines.xhtml

Thymus size assessment

Thymus size of infants was assessed sonographically at 1, 8, 24 and 52 weeks of age using a validated method.[24] The transverse diameter of the thymus and the sagittal area of its largest lobe were detected and multiplied to give a volume-related thymic index (TI). Full details of the thymic index measurements of the ENID trial can be found in Moore *et al.* (2019).[25]

Antibody response to vaccination assessment

All infants were immunized in accordance with the Gambia EPI programme.[23] Three injections of combined DTP were given to children at week 8, 12 and 16. Venous blood samples collected from infants at 12 and 24 weeks and were used to determine the antibody response to vaccination. A multiple immunoassay based on Luminex xMAP technology was used to detect serum-specific IgG antibody responses against diphtheria toxoid, pertussis toxin and tetanus toxin.[26,27] Full details of the outcomes (antibody response to vaccination) of the ENID trial can be found in Okala *et al.* (2019).[28]

Aflatoxin exposure measurement

AF-alb levels in serum taken at 24 and 52 weeks of age were measured at the University of Leeds using an ELISA method [29] that has been previously validated against dietary intake [30]. In brief, this analysis is performed in four steps: albumin extraction and quantification, hydrolysis of albumin with pronase, purification of aflatoxin-albumin residues, and the competitive ELISA. The ELISA involves the pre-mixing of standards, samples, or controls with the rabbit anti aflatoxin-Cl_2-BSA polyclonal antibody, followed by competitive ELISA in which remaining unbound antibody can bind to aflatoxin ovalbumin conjugates coated on the surface of the ELISA plate well. After washing, the bound primary antibody is detected by incubation with an enzyme labelled goat anti-rabbit IgG secondary antibody. Control samples at four known concentrations of AF-alb were examined alongside each batch of samples.

Samples were measured at least twice, in triplicate each time, on two different days. Only results with a coefficient of variation (CV) less than 25% were accepted. The limit of detection (LOD) of the assay was 3 pg/mg albumin. The AF-alb level in samples less than LOD were assigned a value of 1.5 pg/mg for statistical analyses.

Statistical analysis

Statistical analysis was performed using STATA version 15 (StataCorp LP). Distribution of AF-alb level, TI and Ab response to vaccination data were skewed and were therefore log transformed prior to further analysis. Ab fold change was calculated as the Ab level at 24 weeks

For peer review only - http://bmjopen.bmj.com/site/about/guidelines.xhtml

divided by Ab level at 12 weeks. Results were divided into low and high AF exposure groups at both 24 weeks and 52 weeks based on the median of AF-alb level at each time point. Student's *t-test* was used to compare the association of parameters in different seasons or low/high AF exposure level groups.

For other covariates, the season of sampling was categorised as rainy and dry season based on the date of TI measurement and blood sample collection for Ab and AF-alb analysis. There is a distinct seasonal pattern in The Gambia with a period of rainfall from July to October (rainy season) and a long dry season between November and June. The age (months) of introduction of non-breast milk foods to infants were recorded. Infant morbidity was coded as sum of the number of morbidity episodes (diarrhoea, vomiting, cough, rapid breathing and fever).

Relationships between AF-alb level and TI or Ab titre were explored using ordinary least squares regression for individual time points, or random effects model for the pooling data of all time points. Data are presented in different models for TI and Ab titres. The correlation between AF-alb level and TI were analysed as model 1: unadjusted; model 2: adjusted for infant size (length), sex and season at TI measurement; model 3: adjusted for infant size (length), season at TI measurement, sex and maternal supplement groups (for age <= 24 weeks) or both maternal and infants supplement groups (for age at 52 weeks). The correlation between AF-alb level and Ab response were analysed as model 1: unadjusted; model 2: adjusted for weight for height z-score (WHZ) and season of sample collection; model 3: adjusted for WHZ and season of sample collection, sex, maternal supplement groups, haemoglobin (Hb) levels and morbidity.

Ethical approval and consent to participate

Ethical approval for the ENID trial, and the aflatoxin sub-study was obtained from the joint Gambian Government/Medical Research Council (MRC) Unit The Gambia ethics committee (SCC1126v2, L2013.40). Written informed consent was obtained from all participants.

Patient and public involvement

Patients or the public were not involved in the design, conduct, reporting or dissemination plans of our research.

Results

For peer review only - http://bmjopen.bmj.com/site/about/guidelines.xhtml

A total of 800 live born infants were delivered into the main ENID trial.[25] For the current sub-study, 374 infants were included. Table 1 summarizes the characteristics of the participants in the current sub-study. More than half of mothers (67%) had less than one-year of formal education. Twenty-five out of 374 (6.7%) children were born with a low birth weight (<2500g). The mean (SD) duration of exclusive breast feeding was 5.2 (1.3) months.

Table 1. Characteristics of children in current study

**Variable**	**n**	**Mean ± SD / n (%)**
Gender, n (%)	374	
Male		192 (51.3)
Female		182 (48.7)
Ethnicity, n (%)	348	
Fula		30 (8.6)
Jola		11 (3.2)
Mandinka		304 (87.4)
Other		3 (0.9)
Mother's education, n (%)	352	
<1 year formal education		235 (66.8)
>1 year formal education		117 (33.2)
Birth weight (kg)	335	3.0 ± 0.4
Birth length (cm)	335	49.7 ± 2.0
Age of introduction of non-breast milk foods (weeks)		
<6 months	248	4.6 ± 1.3
>6 months	125	6.2 ± 0.1
Morbidity		
sum of first 12 weeks of life	360	8.3 ± 11.0
sum of first 24 weeks of life	361	18.7 ± 18.3
Maternal supplementation group, n (%)	374	
FeFol		95 (25.4)
MMN		96 (25.7)
PE + FeFol		89 (23.7)
PE + MMN		94 (25.1)
Infant supplementation group (6 months), n (%)	374	
LNS+MMN		192 (51.3)
LNS only		182 (48.7)
**AF-alb (pg/mg)**		**GM (95% CI)**
Week 24	352	3.52 (3.15, 3.94)
Week 52	331	25.39 (22.37, 28.82)

For peer review only

Aflatoxin exposure

The geometric mean (GM) level of AF-alb was 3.52 pg/mg at 24 weeks of age and increased to 25.39 pg/mg at 52 weeks with 52% and 2% of samples having levels lower than the LOD (3 pg/mg) at the 24 and 52 weeks age point, respectively. Seasonal variations were observed in the exposure (Figure 1) with around two-fold higher levels of AF-alb determined in samples collected during the dry season compared to the rainy season at both 24 and 52 weeks of age (GM 24wk: 2.5 vs 4.3 pg/mg, $p<0.0001$; 52wk: 19.3 vs 35.5 pg/mg, $p<0.0001$).

Thymic index

Thymus growth showed an upward trend during the first 24 weeks of age (Table 2). A significant increase was shown from birth to 8 weeks of age and reached maximum size at 24 weeks, followed by a decrease by 52 weeks. Seasonal variations were also identified in TI, with the TI values measured during the dry season being slightly higher than those measured in the rainy season at all time points, but this difference was only statistically significant in infants when measured at 8 weeks of age (mean 14.3 vs 13.3 cm^3, $p=0.0136$) (Figure 2).

Table 2. Mean level of thymic index and geometric mean level of antibody response to vaccination in the ENID trial and the current sub-study.

	n	Current sub-study<br>GM (95%CI) / Mean ± SD / (%)[a]	n	ENID trial<br>GM (95%CI) / Mean ± SD / (%)[a]
Thymic index (cm^3)				
Week 1	371	9.21 ± 3.13	765	9.18 ± 3.08
Week 8	369	13.97 ± 4.01	752	13.9 ± 4.09
Week 24	372	14.77 ± 4.25	747	14.7 ± 4.20
Week 52	362	13.59 ± 3.4	707	13.2 ± 3.71
Ab response to vaccination				
Pertussis (EU/ml)				
Week 12	355	7.61 (6.77, 8.57) / 69.3%	711	5.52 (5.04, 6.03) / 50.1%
Week 24	322	132.37 (111.44, 157.22) / 96.0%	663	89.81 (77.71, 103.79) / 88.2%
Diphtheria (IU/ml)				
Week 12	355	0.26 (0.22, 0.32) / 72.4 %	711	0.12 (0.11, 0.14) / 55.5%
Week 24	322	1.18 (1.04, 1.34) / 94.1%	663	1.40 (1.30, 1.51) / 96.8%
Tetanus (IU/ml)				
Week 12	355	0.83 (0.75, 0.91) / 98.9%	711	0.64 (0.59, 0.68) / 97.3%
Week 24	322	4.34 (3.81, 4.94) / 99.7%	663	3.71 (3.40, 4.05) / 99.6%
Morbidity				

For peer review only

Sum of 12 weeks	360	8.3 ± 10.9	729	9.6 ± 12.1
Sum of 24 weeks	361	18.7 ± 18.3	730	22.8 ± 23.5

International standards protective antibody titres (WHO): diphtheria and tetanus >0.1 IU/ml;[24,28] pertussis >5.0 EU/ml.[31] The (%)[a] presented the percentage of samples had protective antibody titres.

Antibody response to DTP vaccination

The Ab response to vaccination increased significantly at 24 weeks of age compared to 12 weeks (Table 2). For pertussis, the protective Ab titres were detected in 69.3% of infants after the first dose, increasing to 96.0% at 24 weeks. For diphtheria, 72.4% of infants at 12 weeks and 94.1% at 24 weeks showed protective levels. For tetanus, the rates were 98.9% and 99.7% between the two time points. Ab titres were slightly higher in samples collected during the rainy season at 12 weeks of age, but lower at 24 weeks of age (Figure 3).

Primary outcomes of ENID trial vs ENID-sub study

Table 2 compares the mean TI value and GM level of Ab titres in our aflatoxin sub-study and the full ENID trial. Infants included in our sub-study showed no significant difference in mean TI when compared with the overall 800 infants in the ENID trial. In terms of the Ab response titres, except for the Ab response to diphtheria at 24 weeks, both the GM of Ab titres and the percentage of samples with protective antibody titres were higher in the sub-group studied here than in the full cohort of ENID infants. However, morbidity was higher among the ENID trial subjects than in our sub-samples at both 12 and 24 weeks.

Aflatoxin exposure and primary outcomes

Figures 2 and 3 present the TI and Ab titre in the low and high AF-alb groups. The mean TI was lower in the high AF-alb group than in the low AF-alb group during the first 24 weeks, but the difference was only statistically significant at 8 weeks (mean 14.9 vs 13.1 cm^3, $p<0.0001$), and not present at 52 weeks (Figure 2). There were no significant differences for Ab response to pertussis and tetanus vaccines between the low and high AF-alb groups (Figure 3). However, a significantly higher Ab titre against diphtheria was determined in infants in the high AF-alb group compared to those in the low AF-alb group at both time points (Ab GM at wk12 : 0.16 vs 0.46 IU/ml $p<0.0001$; Ab GM at wk24 : 0.96 vs 1.46 IU/ml, $p=0.0011$).

Regression analysis with or without adjusting for the other variables showed there were no significant associations between AF-alb level and TI at 1, 24 or 52 weeks (Table 3). However, significant negative correlations were determined between AF-alb and TI at 8 weeks in all

For peer review only - http://bmjopen.bmj.com/site/about/guidelines.xhtml

unadjusted and adjusted models. In addition, the random effect model, which measured the AF-alb correlation with all time points of TI pooled data also showed significantly negative correlations at 24 weeks in both unadjusted and adjusted models, and at 52 weeks in adjusted models.

Table 3. Relationship between aflatoxin-albumin level and thymic index

	Model 1		Model 2		Model 3	
	**Coef. (SE)**	**p**	**Coef. (SE)**	**p**	**Coef. (SE)**	**p**
Week 1	-0.0083 (0.018)	0.637	-0.0012 (0.018)	0.95	-0.0012 (0.018)	0.948
Week 8	-0.0606 (0.014)	<0.001	-0.056 (0.014)	<0.001	-0.056 (0.014)	<0.001
Week 24	-0.0098 (0.014)	0.49	-0.015 (0.015)	0.3	-0.015 (0.015)	0.301
Week 52	-0.0115 (0.014)	0.399	-0.014 (0.014)	0.296	-0.014 (0.014)	0.301
all time points to infant age <24 weeks	-0.027 (0.011)	0.014	-0.023 (0.010)	0.022	-0.023 (0.010)	0.021
all time points to infant age <52 weeks	0.0076 (0.007)	0.28	-0.053 (0.007)	<0.001	-0.052 (0.007)	<0.001

Notes:
TI at 1, 8 and 24 weeks were analysed with AF-alb level at 24 weeks. TI at 52 weeks was analysed with AF-alb level at 52 weeks.
Model 1 unadjusted
Model 2 adjusted for infant size (length), sex and season at TI measurement
Model 3 adjusted for infant size (length), season at TI measurement, sex and maternal supplement groups (for age <= 24 weeks) & infants supplement groups (for age at 52 weeks)

Regression analysis confirmed a significant positive correlation between AF exposure level and Ab response to diphtheria at both 12 and 24 weeks of age ($p<0.001$ & $p=0.002$, respectively), but there were no significant associations between AF-alb level and Ab response to pertussis and tetanus (Table 4).

Neither maternal nor infant supplementation had a significant effect on AF-alb level, TI or Ab titre at any time point (data not presented).

For peer review only - http://bmjopen.bmj.com/site/about/guidelines.xhtml

Table 4. Relationship between aflatoxin-albumin level and antibody response to vaccination

	Model 1		Model 2		Model 3	
	Coef. (SE)	p	Coef. (SE)	p	Coef. (SE)	p
**Pertussis**						
Week 12	-0.093 (0.058)	0.11	-0.093 (0.060)	0.123	-0.088 (0.070)	0.207
Week 24	0.143 (0.083)	0.085	0.133 (0.086)	0.124	0.099 (0.096)	0.301
Fold change	0.190 (0.109)	0.083	0.197 (0.113)	0.084	0.127 (0.127)	0.319
**Diphtheria**						
Week 12	0.421 (0.093)	<0.001	0.418 (0.094)	<0.001	0.495 (0.109)	<0.001
Week 24	0.186 (0.058)	0.002	0.190 (0.061)	0.002	0.204 (0.064)	0.002
Fold change	-0.239 (0.097)	0.014	-0.124 (0.097)	0.204	-0.149 (0.106)	0.162
**Tetanus**						
Week 12	0.065 (0.048)	0.175	0.065 (0.049)	0.182	0.096 (0.056)	0.089
Week 24	-0.034 (0.062)	0.578	-0.064 (0.064)	0.322	-0.071 (0.070)	0.316
Fold change	-0.111 (0.081)	0.172	-0.096 (0.084)	0.252	-0.127 (0.092)	0.168

Notes:
Antibody response to vaccination were analysed with AF-alb level at 24 weeks only.
Model 1 unadjusted
Model 2 adjusted for WHZ and season of sample collection
Model 3 adjusted for WHZ and season of sample collection, sex, maternal supplement group, Hb levels and morbidity.

For peer review only

Discussion

This is the first study to investigate the impact of aflatoxin exposure on immune function of infants less than 12 months of age using TI and antibody response to DTP vaccination as indicators of immune function. Infants who were exposed to higher levels of aflatoxin, had smaller thymus during the first 24 weeks of age, and had significantly higher protective Ab titres against diphtheria.

The observed high prevalence of AF exposure in the present study is consistent with previous findings in the same region of Gambia.[5,32,33] It is worth noting that the ELISA method used

For peer review only - http://bmjopen.bmj.com/site/about/guidelines.xhtml

in this study gives AF-alb values that are approximately three-fold higher than aflatoxin lysine values measured by LC-MS methods but that results between methods correlate for the same samples [34]. This issue was discussed by McMillan et al [11] and needs to be taken into consideration when comparing values obtained using the different methods. An increasing prevalence and upward trend of AF-alb levels with age were determined in our study. Infants at 52 weeks showed more than seven-fold higher levels of AF-alb than at 24 weeks of age. Aflatoxin exposure could happen *in utero* through trans-placental exposure, and the exposure level will increase during the first year when infants are gradually introduced to complementary foods.[2]

The Gambia has pronounced dry and rainy seasons, and previous studies have shown a seasonal impact on both growth and aflatoxin exposure.[6,35] In the present study, serum samples collected during the dry season had significantly higher levels of AF-alb than those samples collected during the rainy season. The annual dry season in The Gambia is a time of relative food availability, as food supplies from the previous harvest are usually plentiful. Aflatoxin contamination in foods and crops tends to increase after a period of storage.[36] Populations are more likely to consume old grains towards the end of the dry (harvest) season, and foods which have been stored for a period are more likely to be contaminated. Our findings are consistent with previous studies in Benin and Guinea.[10,37] However, exposure can also depend on food type. In Senegal, Watson *et al.* found that higher AF-alb levels in the harvest season compared to the post-harvest season correlated with the recorded high consumption (four or more days a week) of contaminated groundnuts during the harvest period, with groundnuts being more susceptible to contamination than maize.[38]

Similar growth trend and seasonal variation of TI were previously reported in Gambia by Collinson et al. who also reported the consistently smaller thymus size in the rainy season, and significant difference at 8 weeks of age (p=0.001).[39] The significant retardation of thymus at 8 weeks found in children with high aflatoxin exposure might due to it is the time point that the thymus grows fastest. The sonographic method used in the current study to assess TI only represents an anatomical feature and not function, but previous studies in animals and infants have demonstrated a correlation between thymus size and lymphocyte proportion and function [40,41]. Therefore, smaller TI in infants could predict a lower immunity in the future. Previously, AF induced damage on the thymus has only been investigated in animal models. A recent study reported that AF caused thymic histopathological lesions and pathological impairments in chickens which had been fed with aflatoxin contaminated feeds (34.3–134 μg

For peer review only

AFB_1/kg corn feed) for 21 and 42 days.[41] The reduction in thymus size and number of apoptotic lymphocytes occurred in a dose-dependent manner.

There was no previous study investigating the influence of aflatoxin exposure on Ab response to DTP vaccination. In our study, we found a significant positive association between aflatoxin biomarker level and Ab titre to diphtheria. An earlier study conducted in Gambian infants determined a similar weak but significant positive correlation between AF-alb level and one serotype of anti-pneumococcal Ab.[15] An animal study to induce anti-aflatoxin B_1 antibody in dairy cows which combined AFB_1 with recombinant diphtheria toxin molecules injected into heifers boosted the generation of anti-AFB_1 Abs.[42] The effect of AF on Ab response could vary depending on the feature of the vaccines, as early animal studies conducted in chicken and rabbits reported inverse influence of AF exposure level on Ab response to different vaccines.[43,44] The mechanism behind the significant positive correlation between AF-alb level and Ab titre to diphtheria needs further investigation.

One limitation of the current study was the AF-alb levels were not measured at the birth of infants or in the maternal blood, so whilst the AF-alb biomarker integrates exposure over the previous 2-3 months,[45] AF-alb levels may not accurately reflect exposure in early weeks. However, and Turner *et al.* [5] reported a close association of AF-alb levels in maternal and infants' cord blood, and also reported a significant negative correlation between maternal AF biomarker level and child weight and height gain at the first year of life.[5] In our study, we found the TI was significantly correlated with the weight of infants in the first year. Considering the AF associated growth retardation determined in infants in the current cohort,[7] we suggest that AF could be a potential factor that influenced child growth and thymus development during the early stages of life.

A few previous studies have examined the effect of high levels of aflatoxin exposure on markers of immunity in human. Turner *et al.* (2003) reported a decrease in expression of salivary IgA in children from rural Gambia with detectable AF-alb levels compared with those with undetectable levels.[15] A lower percentage of T and B cells have been observed in participants with high concentrations of AF-alb in Ghanaians aged between 19 and 86 years old.[16,17] However, some animal studies of aflatoxin induced effects on the immune system are inconsistent: Li *et al.* (2014) and Meissonnier *et al.* (2008) found no significant effect of aflatoxin B_1 on humoral immunity function in chickens and pigs.[46,47] While another study found increased expression of IgM and IgG in pigs dosed with high AF contaminated feed.[48] The mechanism of the immunoglobulin rise is unclear.

For peer review only - http://bmjopen.bmj.com/site/about/guidelines.xhtml

In conclusion, we have demonstrated a significant effect of aflatoxin exposure on infant immune development, assessed by thymic size. Less consistent evidence was found for infants' antibody response to vaccination. This study adds to evidence that aflatoxin exposure in infants can modify the immune response, and further efforts should be made to ameliorate dietary aflatoxin exposure across populations in sub-Saharan Africa.

Funding statement

The research reported here was funded by the Meridian Institute (Agreement Number 9678.0) and the Bill and Melinda Gates Foundation (grant no: OPP1066947). The ENID trial was supported by the UK Medical Research Council (MRC) (MC-A760-5QX00) and the UK Department for International Development (DFID) under the MRC/ DFID Concordat agreement. The dataset analysed in this paper is available from the corresponding author on reasonable request, and with appropriate additional ethical approvals, where necessary.

Contributors

MNR, YYG, AMP and SEM contributed to the original study design and implementation. YX, GC, PN and TF have contributed to laboratory analysis. YX wrote the first draft of the manuscript. All authors approved the final version of the manuscript.

Competing Interest statement

The authors declare no competing interests.

Data sharing statement

Data not included in the manuscript are available upon reasonable request

References

1 Gong YY, Watson S, Routledge MN. Aflatoxin Exposure and Associated Human Health Effects, a Review of Epidemiological Studies. *Food Saf* 2016;4:14–27. doi:10.14252/foodsafetyfscj.2015026

2 Gong YY, Egal S, Hounsa A, *et al.* Determinants of aflatoxin exposure in young children from Benin and Togo, West Africa: The critical role of weaning. *Int J Epidemiol* 2003;32:556–62.

For peer review only - http://bmjopen.bmj.com/site/about/guidelines.xhtml

doi:10.1093/ije/dyg109

3 Hudson GJ, Wild CP, Zarba A, *et al.* Aflatoxins isolated by immunoaffinity chromatography from foods consumed in the Gambia, West Africa. *Nat Toxins* 1992;1:100–5. doi:10.1002/nt.2620010208

4 Wild P, Whittle C, Hall J, *et al.* Liver Enzymes , and Hepatitis Gambian B Virus Infection in. 1993;2:555–62.

5 Turner PC, Collinson AC, Cheung YB, *et al.* Aflatoxin exposure in utero causes growth faltering in Gambian infants. *Int J Epidemiol* 2007;36:1119–25. doi:10.1093/ije/dym122

6 Castelino JM, Dominguez-Salas P, Routledge MN, *et al.* Seasonal and gestation stage associated differences in aflatoxin exposure in pregnant Gambian women. *Trop Med Int Heal* 2014;19:348–54. doi:10.1111/tmi.12250

7 Watson S, Moore SE, Darboe MK, *et al.* Impaired growth in rural Gambian infants exposed to aflatoxin: A prospective cohort study. *BMC Public Health* 2018;18 doi:10.1186/s12889-018-6164-4

8 IARC. Some traditional herbal medicines, some mycotoxins, naphthalene and styrene. *IARC Monogr Eval Carcinog Risks Hum* 2002;82:1–556. doi:10.1002/food.19940380335

9 Gong YY, Cardwell K, Hounsa A, *et al.* Dietary aflatoxin exposure and impaired growth in young children from Benin and Togo: Cross sectional study. *Br Med J* 2002;325:20–1. doi:10.1136/bmj.325.7354.20

10 Gong Y, Hounsa A, Egal S, *et al.* Postweaning exposure to aflatoxin results in impaired child growth: A longitudinal study in Benin, West Africa. *Environ Health Perspect* 2004;112:1334–8. doi:10.1289/ehp.6954

11 McMillan A, Renaud JB, Burgess KMN *et al.* Aflatoxin exposure in Nigerian children with severe acute malnutrition Food and Chemical Toxicology 2018;111:356-362.

12 Wangia-Dixon RN, Xue KS, Alcala J *et al.* Nutrition and growth outcomes are affected by aflatoxin exposures in Kenyan children. Food Additives and Contaminants Part A 2020;37:2123-2134.

13 Bbosa GS, Kitya D, Lubega A, *et al.* Review of the Biological and Health Effects of Aflatoxins on Body Organs and Body Systems. In: *Aflatoxins - Recent Advances and Future Prospects*. 2013. doi:10.5772/51201

14 Wild C, Miller J, Groopman J. *Mycotoxin Control in Low- and Middle- Income Countries*. 2015.

For peer review only

http://www.ncbi.nlm.nih.gov/pubmed/27030861

15 Turner PC, Moore SE, Hall AJ, *et al.* Modification of immune function through exposure to dietary aflatoxin in Gambian children. *Environ Health Perspect* 2003;111:217–20. doi:10.1289/ehp.5753

16 Jiang Y, Jolly PE, Ellis WO, *et al.* Aflatoxin B1 albumin adduct levels and cellular immune status in Ghanaians. *Int Immunol* 2005;17:807–14. doi:10.1093/intimm/dxh262

17 Jiang Y, Jolly PE, Preko P, *et al.* Aflatoxin-related immune dysfunction in health and in human immunodeficiency virus disease. *Clin Dev Immunol* 2008;790309. doi:10.1155/2008/790309

18 Aaby P, Marx C, Trautner S, *et al.* Thymus size at birth is associated with infant mortality: A community study from Guinea-Bissau. *Acta Paediatr Int J Paediatr* 2002;91:698–703. doi:10.1080/080352502760069142

19 Garly ML, Trautner SL, Marx C, *et al.* Thymus Size at 6 Months of Age and Subsequent Child Mortality. *J Pediatr* 2008;153. doi:10.1016/j.jpeds.2008.04.069

20 Moore SE, Fulford AJC, Wagatsuma Y, *et al.* Thymus development and infant and child mortality in rural Bangladesh. *Int J Epidemiol* 2014;43:216–23. doi:10.1093/ije/dyt232

21 Bondy GS, Pestka JJ. Immunomodulation by fungal toxins. *J Toxicol Environ Heal - Part B Crit Rev* Published Online First: 2000. doi:10.1080/109374000281113

22 Meissonnier GM, Marin DE, Galtier P, *et al.* Modulation of the immune response by a group of fungal food contaminant, the aflatoxins. *Nutr Immun* 2006.

23 Moore SE, Fulford AJC, Darboe MK, *et al.* A randomized trial to investigate the effects of pre-natal and infant nutritional supplementation on infant immune development in rural Gambia: The ENID trial: Early Nutrition and Immune Development. *BMC Pregnancy Childbirth* 2012;12:1–8. doi:10.1186/1471-2393-12-107

24 Hasselbalch H, Nielsen MB, Jeppesen D, *et al.* Sonographic measurement of the thymus in infants. *Eur Radiol* 1996;6:700–3. doi:10.1007/BF00187675

25 Moore SE, Fulford AJC, Sosseh F, *et al.* Thymic size is increased by infancy, but not pregnancy, nutritional supplementation in rural Gambian children: A randomized clinical trial. *BMC Med* 2019;17:1–9. doi:10.1186/s12916-019-1264-2

26 Colombet I, Saguez C, Sanson-Le Pors MJ, *et al.* Diagnosis of tetanus immunization status: Multicenter assessment of a rapid biological test. *Clin Diagn Lab Immunol* 2005;12:1057–62.

For peer review only

doi:10.1128/CDLI.12.9.1057-1062.2005

27 van Gageldonk PGM, van Schaijk FG, van der Klis FR, *et al.* Development and validation of a multiplex immunoassay for the simultaneous determination of serum antibodies to Bordetella pertussis, diphtheria and tetanus. *J Immunol Methods* Published Online First: 2008. doi:10.1016/j.jim.2008.02.018

28 Okala SG, Darboe MK, Sosseh F, *et al.* Impact of nutritional supplementation during pregnancy on antibody responses to diphtheria-tetanus-pertussis vaccination in infants: A randomised trial in the Gambia. *PLoS Med* 2019;16. doi:10.1371/journal.pmed.1002854

29 Chapot B, Wild C. *ELISA for quantification of aflatoxin-albumin adducts and their application to human exposure assessment*. Academic Press, San Diego 1991.

30 Routledge MN, Kimanya ME, Shirima CP, *et al.* Quantitative correlation of aflatoxin biomarker with dietary intake of aflatoxin in Tanzanian children. *Biomarkers* 2014;19:430-435.

31 van Gageldonk PGM, van Schaijk FG, van der Klis FR, *et al.* Development and validation of a multiplex immunoassay for the simultaneous determination of serum antibodies to Bordetella pertussis, diphtheria and tetanus. *J Immunol Methods* 2008;335:79–89. doi:10.1016/j.jim.2008.02.018

32 Turner PC, Mendy M, Whittle H, *et al.* Hepatitis B infection and aflatoxin biomarker levels in Gambian children. *Trop Med Int Heal* 2000;5:837–41. doi:10.1046/j.1365-3156.2000.00664.x

33 Wild CP, Yin F, Turner PC, *et al.* Environmental and genetic determinants of aflatoxin-albumin adducts in The Gambia. *Int J Cancer* 2000;86:1–7. doi:10.1002/(SICI)1097-0215(20000401)86:1<1::AID-IJC1>3.0.CO;2-I

34 McCoy LF, Scholl PF, Sutcliffe AE *et al.* Human aflatoxin albumin adducts quantitatively compared by ELISA, HPLC with fluourescence detection, and HPLC with isotope dilution mass spectrometry. Cancer Epi Biom Prev 2008;17:1653-1657.

35 Nabwera HM, Fulford AJ, Moore SE, *et al.* Growth faltering in rural Gambian children after four decades of interventions: a retrospective cohort study. *Lancet Glob Heal* 2017;5:e208–16. doi:10.1016/S2214-109X(16)30355-2

36 Pitt JI, Taniwaki MH, Cole MB. Mycotoxin production in major crops as influenced by growing, harvesting, storage and processing, with emphasis on the achievement of Food Safety Objectives. *Food Control* 2013;32:205–15. doi:10.1016/j.foodcont.2012.11.023

For peer review only

37 Turner PC, Sylla A, Gong YY, *et al.* Reduction in exposure to carcinogenic aflatoxins by postharvest intervention measures in west Africa: A community-based intervention study. *Lancet* 2005;365:1950–6. doi:10.1016/S0140-6736(05)66661-5

38 Watson S, Diedhiou PM, Atehnkeng J, *et al.* Seasonal and geographical differences in aflatoxin exposures in Senegal. *World Mycotoxin J* 2015;8:525–31. doi:10.3920/WMJ2014.1824

39 Collinson AC, Moore SE, Cole TJ, *et al.* Birth season and environmental influences on patterns of thymic growth in rural Gambian infants. *Acta Paediatr Int J Paediatr* 2003;92:1014–20. doi:10.1080/08035250310004379

40 Jeppesen DL, Hasselbalch H, Lisse IM, *et al.* T-lymphocyte subsets, thymic size and breastfeeding in infancy. *Pediatr Allergy Immunol* 2004;15:127–32. doi:10.1111/j.1399-3038.2004.00032.x

41 Peng X, Bai S, Ding X, *et al.* Pathological impairment, cell cycle arrest and apoptosis of thymus and bursa of fabricius induced by aflatoxin-contaminated corn in broilers. *Int J Environ Res Public Health* 2017;14. doi:10.3390/ijerph14010077

42 Giovati L, Gallo A, Masoero F, *et al.* Vaccination of heifers with anaflatoxin improves the reduction of aflatoxin B1 carry over in milk of lactating dairy cows. *PLoS One* 2014;9. doi:10.1371/journal.pone.0094440

43 Azzam AH, Gabal MA. Interaction of aflatoxin in the feed and immunization against selected infectious diseases. I. Infectious bursal disease. *Avian Pathol* 1997;26:317–25. doi:10.1080/03079459708419214

44 Dimitri RA, Gabal MA. Immunosuppressant activity of aflatoxin ingestion in rabbits measured by response to mycobacterium bovis antigen I. Cell mediated immune response measured by skin test reaction. *Vet Hum Toxicol* 1996;38:333–6.

45 Wild CP, Hasegawa R, Barraud L, *et al.* Aflatoxin-albumin adducts: a basis for comparative carcinogenesis between animals and humans. *Cancer Epidemiol Biomarkers Prev* 1996;5:179–89.http://www.ncbi.nlm.nih.gov/pubmed/8833618

46 Li Y, Ma QG, Zhao LH, *et al.* Effects of lipoic acid on immune function, the antioxidant defense system, and inflammation-related genes expression of broiler chickens fed aflatoxin contaminated diets. *Int J Mol Sci* 2014;15:5649–62. doi:10.3390/ijms15045649

47 Meissonnier GM, Pinton P, Laffitte J, *et al.* Immunotoxicity of aflatoxin B1: Impairment of the cell-mediated response to vaccine antigen and modulation of cytokine expression. *Toxicol*

For peer review only

For peer review only - http://bmjopen.bmj.com/site/about/guidelines.xhtml

Appl Pharmacol 2008;231:142–9. doi:10.1016/j.taap.2008.04.004

48 Panangala VS, Giambrone JJ, Diener UL, *et al.* Effects of aflatoxin on the growth performance and immune responses of weanling swine. *Am J Vet Res* 1986;47:2062–7.

Figure Legends

Figure 1. Aflatoxin-albumin level (log transformed) in rainy and dry season at 24 and 52 weeks.

Figure 2. Thymic index in children in low or high Aflatoxin-albumin group or in different seasons. The low and high AF-alb group was divided by the median level at each time point. TI at week 8, 12 and 24 were group by AF-alb at week 24, TI at 52 weeks was group by AF-alb level at week 52. The season of TI was defined as the time of measurement conducted.

Figure 3. Antibody level to vaccination in low or high aflatoxin-albumin group and in different seasons. The low and high AF-alb group was divided by the median level at week 24. The season of Ab level was defined as the date of blood sample collection.

For peer review only

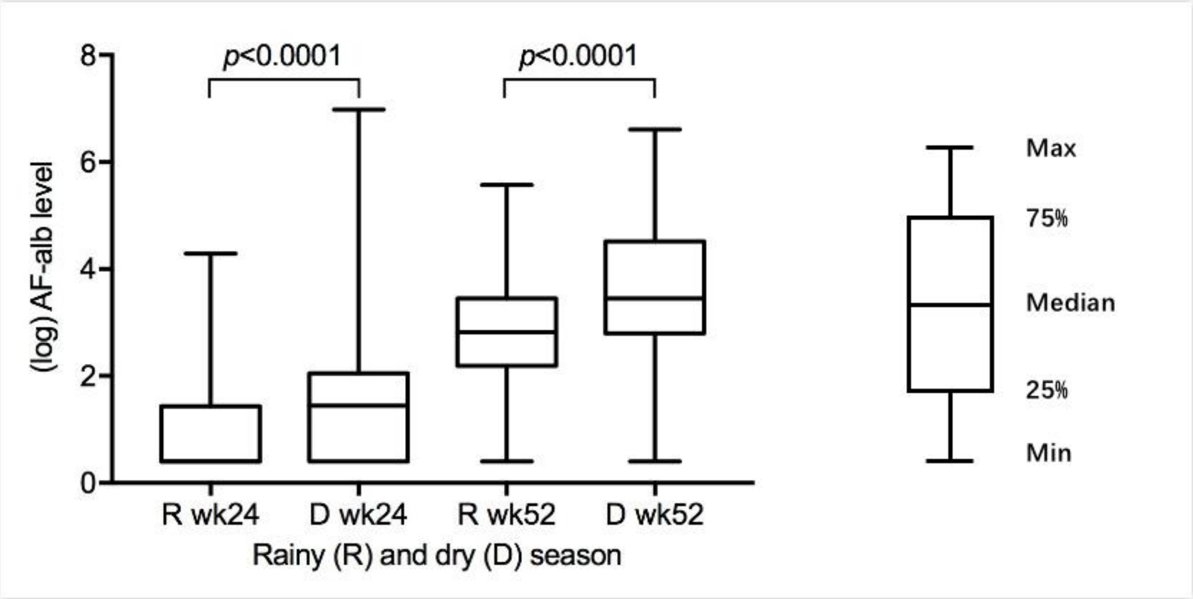

peer review only

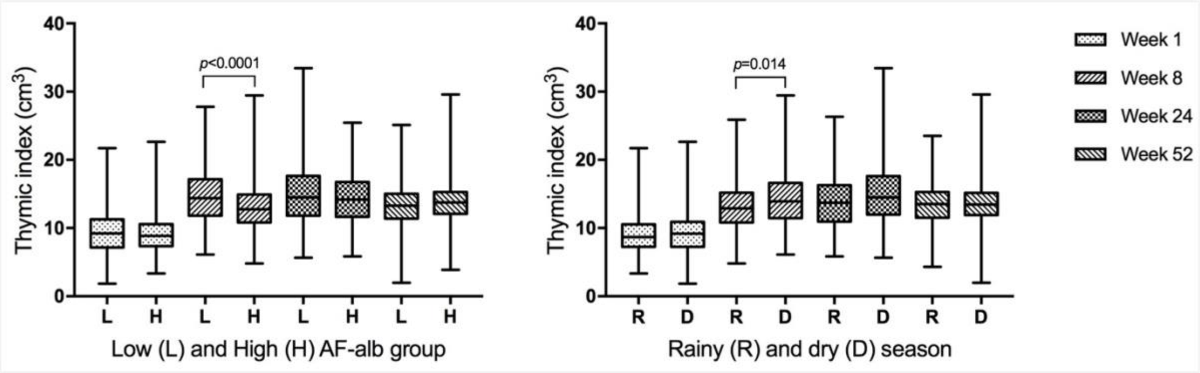

For peer review only

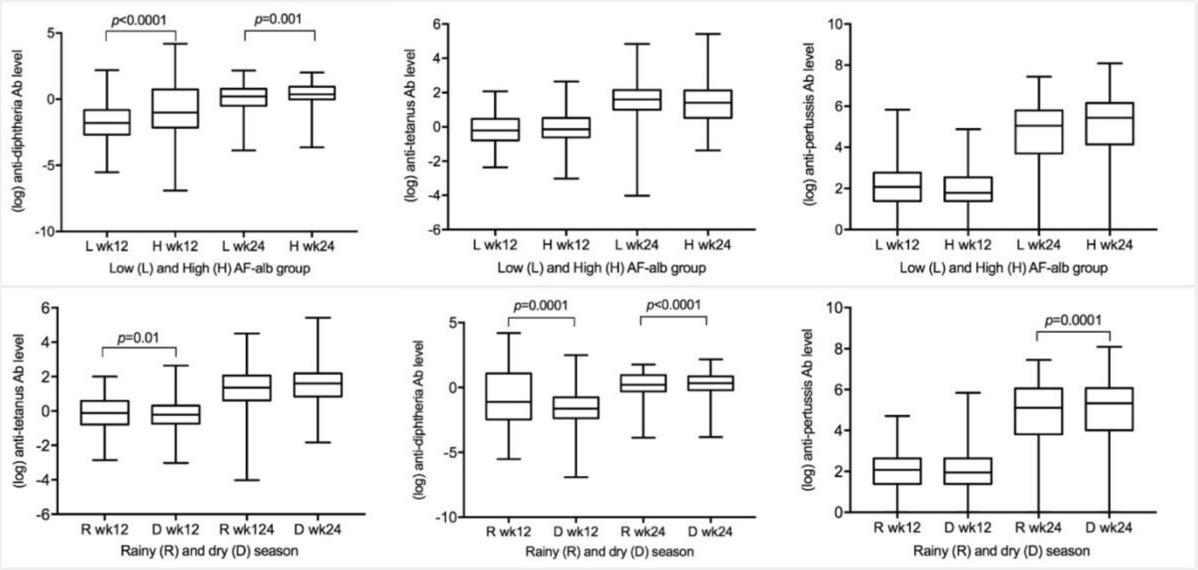

peer review only

STROBE Statement—Checklist of items that should be included in reports of ***cohort studies***

	Item No	Recommendation
**Title and abstract**	1	(*a*) Indicate the study's design with a commonly used term in the title or the abstract **p.1 line 2**
		(*b*) Provide in the abstract an informative and balanced summary of what was done and what was found **p.1 line 16 to p.2 line 5**
**Introduction**		
Background/rationale	2	Explain the scientific background and rationale for the investigation being reported **p.3, lines 1 to 32**
Objectives	3	State specific objectives, including any prespecified hypotheses **p.4 lines 1-2**
**Methods**		
Study design	4	Present key elements of study design early in the paper **p.1 lines 19-21**
Setting	5	Describe the setting, locations, and relevant dates, including periods of recruitment, exposure, follow-up, and data collection. **P.4 lines 6-13 and 24-25.**
Participants	6	(*a*) Give the eligibility criteria, and the sources and methods of selection of participants. Describe methods of follow-up **p.4 line 8, lines 12-14, p.5 lines 1-12.**
		(*b*) For matched studies, give matching criteria and number of exposed and unexposed
Variables	7	Clearly define all outcomes, exposures, predictors, potential confounders, and effect modifiers. Give diagnostic criteria, if applicable **p.5 lines 31-34, p.6 lines 4-9.**
Data sources/ measurement	8*	For each variable of interest, give sources of data and details of methods of assessment (measurement). Describe comparability of assessment methods if there is more than one group **p. 5, lines 1-28.**
Bias	9	Describe any efforts to address potential sources of bias **p.4 lines 12-14 and 20-23.**
Study size	10	Explain how the study size was arrived at **p. 4, line 14**
Quantitative variables	11	Explain how quantitative variables were handled in the analyses. If applicable, describe which groupings were chosen and why
Statistical methods	12	(*a*) Describe all statistical methods, including those used to control for confounding **p.5 line 31 to p.6 line 21**
		(*b*) Describe any methods used to examine subgroups and interactions
		(*c*) Explain how missing data were addressed
		(*d*) If applicable, explain how loss to follow-up was addressed
		(*e*) Describe any sensitivity analyses
**Results**		
Participants	13*	(a) Report numbers of individuals at each stage of study—eg numbers potentially eligible, examined for eligibility, confirmed eligible, included in the study, completing follow-up, and analysed **p 4. Line 14**
		(b) Give reasons for non-participation at each stage
		(c) Consider use of a flow diagram
Descriptive data	14*	(a) Give characteristics of study participants (eg demographic, clinical, social) and information on exposures and potential confounders **p 7, table 1**
		(b) Indicate number of participants with missing data for each variable of interest
		(c) Summarise follow-up time (eg, average and total amount)
Outcome data	15*	Report numbers of outcome events or summary measures over time
Main results	16	(*a*) Give unadjusted estimates and, if applicable, confounder-adjusted estimates and their precision (eg, 95% confidence interval). Make clear which confounders were adjusted for and why they were included **p.10 table 3, p. 11 table 4**

For peer review only - http://bmjopen.bmj.com/site/about/guidelines.xhtml

		(*b*) Report category boundaries when continuous variables were categorized
		(*c*) If relevant, consider translating estimates of relative risk into absolute risk for a meaningful time period
Other analyses	17	Report other analyses done—eg analyses of subgroups and interactions, and sensitivity analyses
**Discussion**		
Key results	18	Summarise key results with reference to study objectives **p.11, lines 12-14**
Limitations	19	Discuss limitations of the study, taking into account sources of potential bias or imprecision. Discuss both direction and magnitude of any potential bias **p.13, lines 12-14.**
Interpretation	20	Give a cautious overall interpretation of results considering objectives, limitations, multiplicity of analyses, results from similar studies, and other relevant evidence **p. 13, line 32 to p. 14 line 2.**
Generalisability	21	Discuss the generalisability (external validity) of the study results **p.13 lines 22-31.**
**Other information**		
Funding	22	Give the source of funding and the role of the funders for the present study and, if applicable, for the original study on which the present article is based **p.14, lines 6-10.**

*Give information separately for exposed and unexposed groups.

Note: An Explanation and Elaboration article discusses each checklist item and gives methodological background and published examples of transparent reporting. The STROBE checklist is best used in conjunction with this article (freely available on the Web sites of PLoS Medicine at http://www.plosmedicine.org/, Annals of Internal Medicine at http://www.annals.org/, and Epidemiology at http://www.epidem.com/). Information on the STROBE Initiative is available at http://www.strobe-statement.org.

For peer review only
